# Supplementary material for: Recommended approaches in the application of toxicogenomics to derive points of departure for chemical risk assessment
Source: Arch Toxicol. 2016 Dec 7;91(5):2045–65. doi: 10.1007/s00204-016-1886-5 (PMC5399047; doi:10.1007/s00204-016-1886-5)
Supplement: Supplementary file 1 — Supplementary material 1 (DOCX 9749 kb) [file 204_2016_1886_MOESM1_ESM.docx]

**SUPPLEMENTARY FIGURE LEGENDS**

**Figure S1.** Box and whisker plots of the BMD_t_ median for the 11 approaches for all chemicals at the 5, 14, 28 and 90 day time points. Colored horizontal lines represent the NOAEL (blue line) and LOAEL (red line). The box boundaries and lines represent the interquartile ranges and medians, respectively. The whiskers represent 10 and 90 percentiles.

**Figure S2.** BMD modeling of continuous data (e.g., organ weight) and dichotomous data (e.g., hypertrophy, hyperplasia, and vacuolation) for the apical endpoint with the lowest BMD_a_ at each time point for TBB, BB and TCP.

**Figure S3.** BMD modeling of continuous data (e.g., organ weight) and dichotomous data (e.g., hypertrophy, hyperplasia, and vacuolation) for the apical endpoint with the lowest BMD_a_ at each time point for MDA, NDPA and HZB.

**Figure S4.** Box and whisker plots of the BMDL_t_ mean for the 11 approaches for all of the chemicals at the 5, 14, 28 and 90 day time points. Colored horizontal lines represent the NOAEL (blue line), LOAEL (red line), lowest time-point matched BMD_a_ value (gray line), the lowest overall BMD_a_ values (i.e., any time) for apical endpoint across all time points (green line), and cancer (black line). The box boundaries and lines represent the interquartile ranges and means, respectively. The whiskers represent 10 and 90 percentiles.

**Figure S5.** BMD_t_s relative to apical PODs for the 14, 28, and 90 day time points. Three-fold and ten-fold ranges from the apical POD are within the shaded areas and dashed horizontal lines, respectively. The BMD_t_s derived from each approach were divided by the POD values for each approach for every chemical.

**Figure S6.** BMD_t_s relative to apical PODs for the 14, 28, and 90 day time points. Data from Figure S5 are shown separately for each chemical.

**Figure S7.** BMDL_t_s relative to NOAEL and LOAEL for 14, 28, and 90 days. Three-fold and ten-fold ranges from the apical POD are within shaded areas and dashed horizontal lines, respectively. The BMD_t_s derived from each approach were divided by the POD values for each approach for every chemical.

**Figure S8.** BMDL_t_s relative to apical PODs for the 14, 28, and 90 day time points. Data from Figure S7 are shown separately for each chemical.

**Figure S9.** BMD_a_ values for apical responses.

Note: pathological changes related to HZB were observed at the 90 day time point only.

**Figure S10.** BMD_a_ at day 5 relative to the lowest apical endpoint across any time point.

**Figure S11.** Correlation between log-transformed BMD_t_ (panel A) and BMDL_t_ (panel B) values derived from the 11 approaches at the 5 day time point and log-transformed POD values. The 1:1 line is indicated as a solid red line, and the 95% confidence interval of the regression line is indicated by dashed blue lines.

**Figure S12.** Correlation between log-transformed BMD_t_ (panel A) and BMDL_t_ (panel B) values derived from the 11 approaches at the 14 day time point and log-transformed POD values. The 1:1 line is indicated by a solid red line, and the 95% confidence interval of the regression line is indicated by dashed blue lines.

**Figure S13.** Correlation between log-transformed BMD_t_ (panel A) and BMDL_t_ (panel B) values derived from the 11 approaches at the 28 day time point and log-transformed POD values. The 1:1 line is indicated by a solid red line, and the 95% confidence interval of the regression line is indicated by dashed blue lines.

**Figure S14.** Correlation between log-transformed BMD_t_ (panel A) and BMDL_t_ (panel B) values derived from the 11 approaches at the 90 day time point and log-transformed POD values. The 1:1 line is indicated by a solid red line, and the 95% confidence interval of the regression line is indicated by dashed blue lines.

**Figure S15.** Correlation between log-transformed mean (top row) and median (bottom row) BMD_t_ values derived from Approach 9 in the current study^1^ at the 5 day, 14 day, 28 day, 90 day, and all time points and log-transformed median BMD_t_ values from Thomas et al (2013)^2^.

**Figure S16.**  ***(A)*** BMD_t_ values derived from the 11 approaches for MDA, NDPA and HZB for 5, 14, 28, and 90 days relative to the cancer BMD_a_ values for incidences of tumor development. Three-fold and ten-fold differences from the apical POD are shown within the shaded areas and dashed horizontal lines, respectively. The BMD_t_s derived from each approach were divided by the BMD_a_ values for every chemical. ***(B)*** Data from panel A are shown separately for each chemical.

**SUPPLEMENTARY MATERIAL**

**Figure S1.**

**
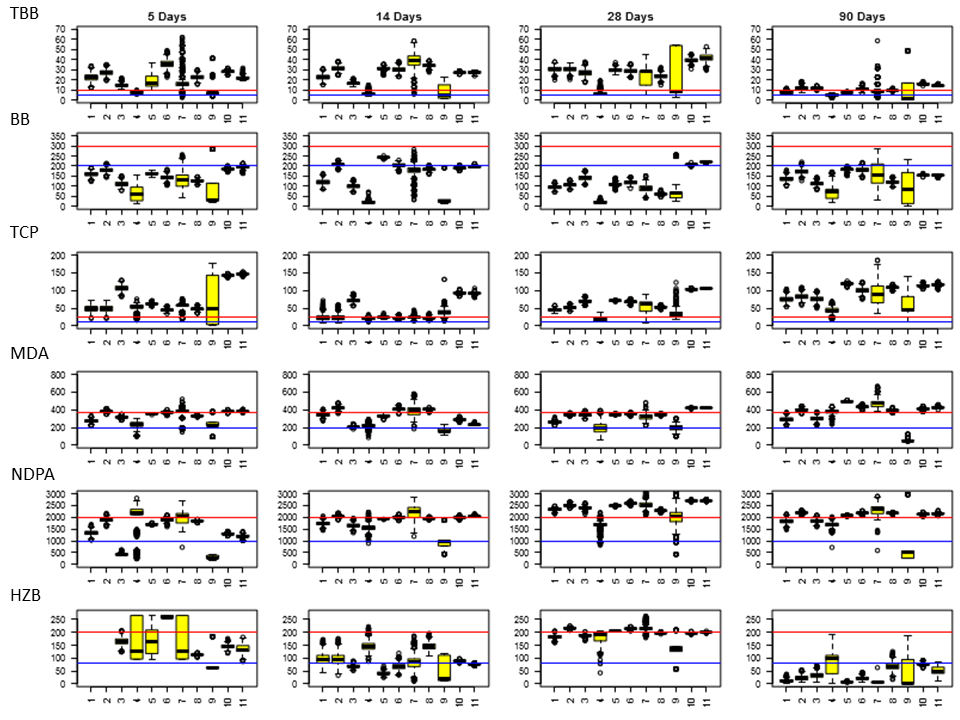
**

**Figure S2.**

**Figure S3.**

**Figure S4.**

**
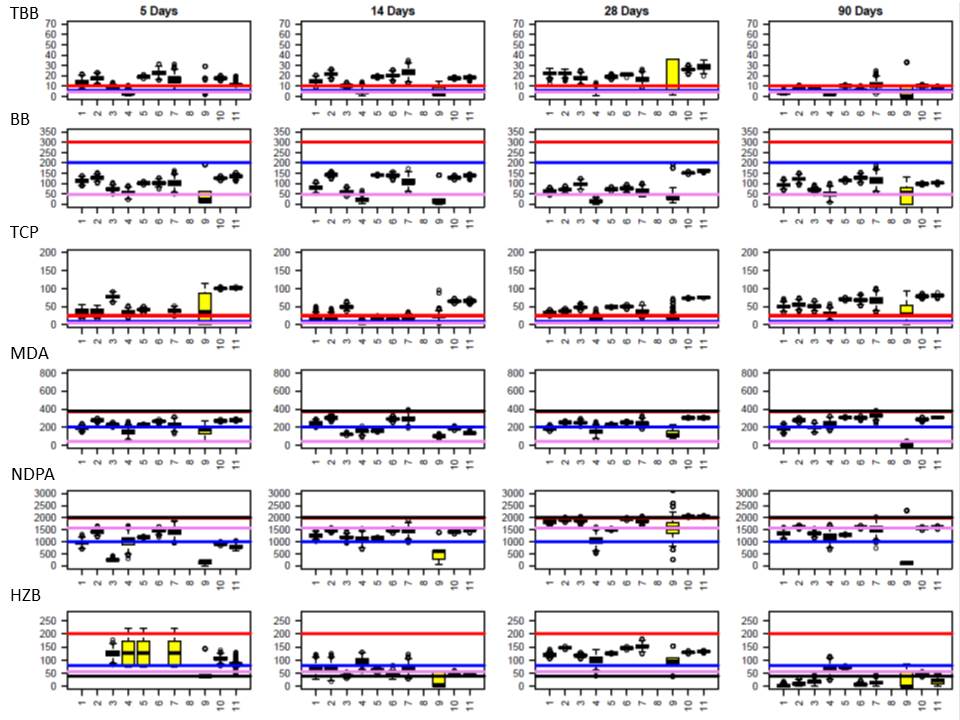
**

**Figure S5.**


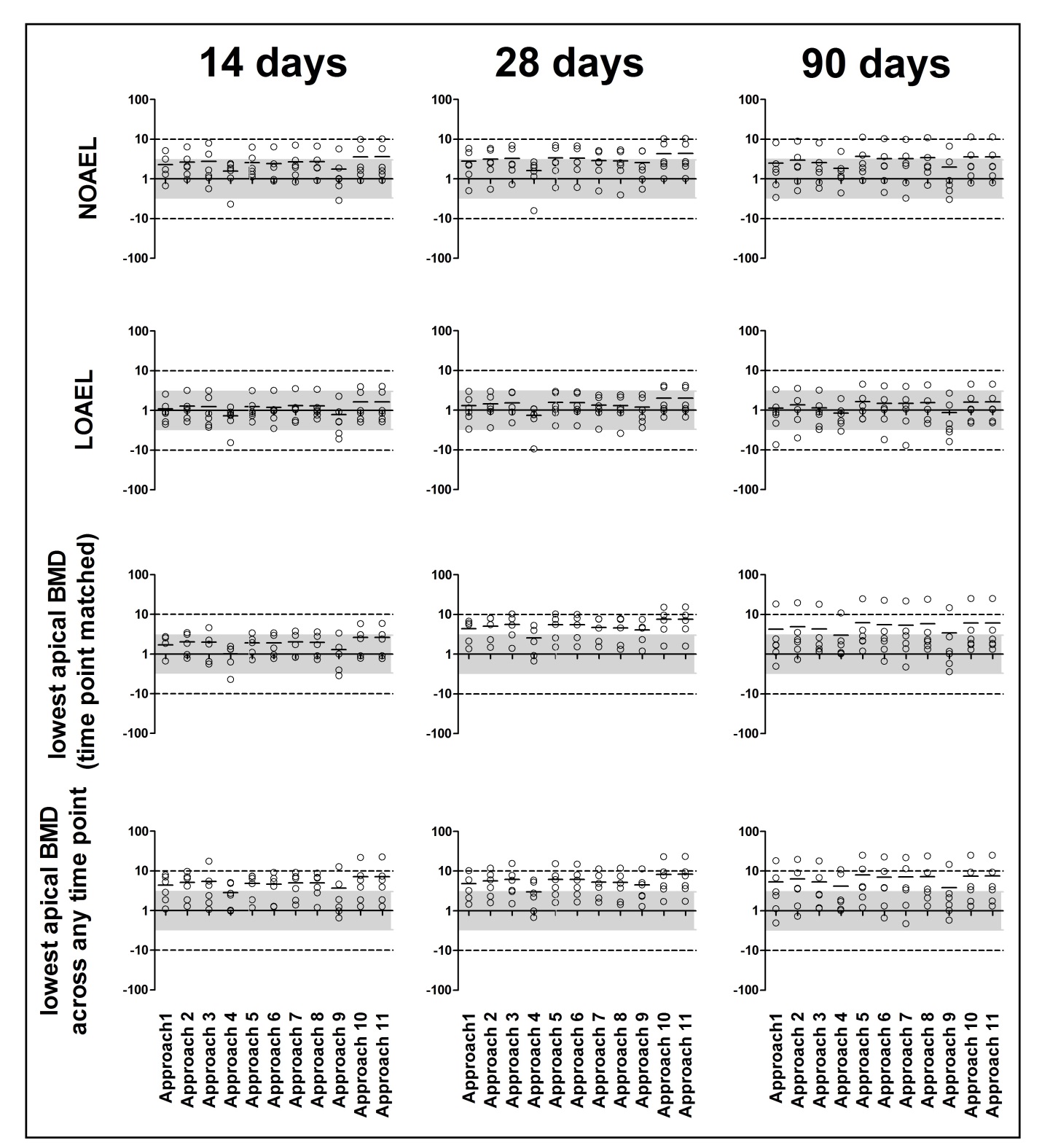


**Figure S6**


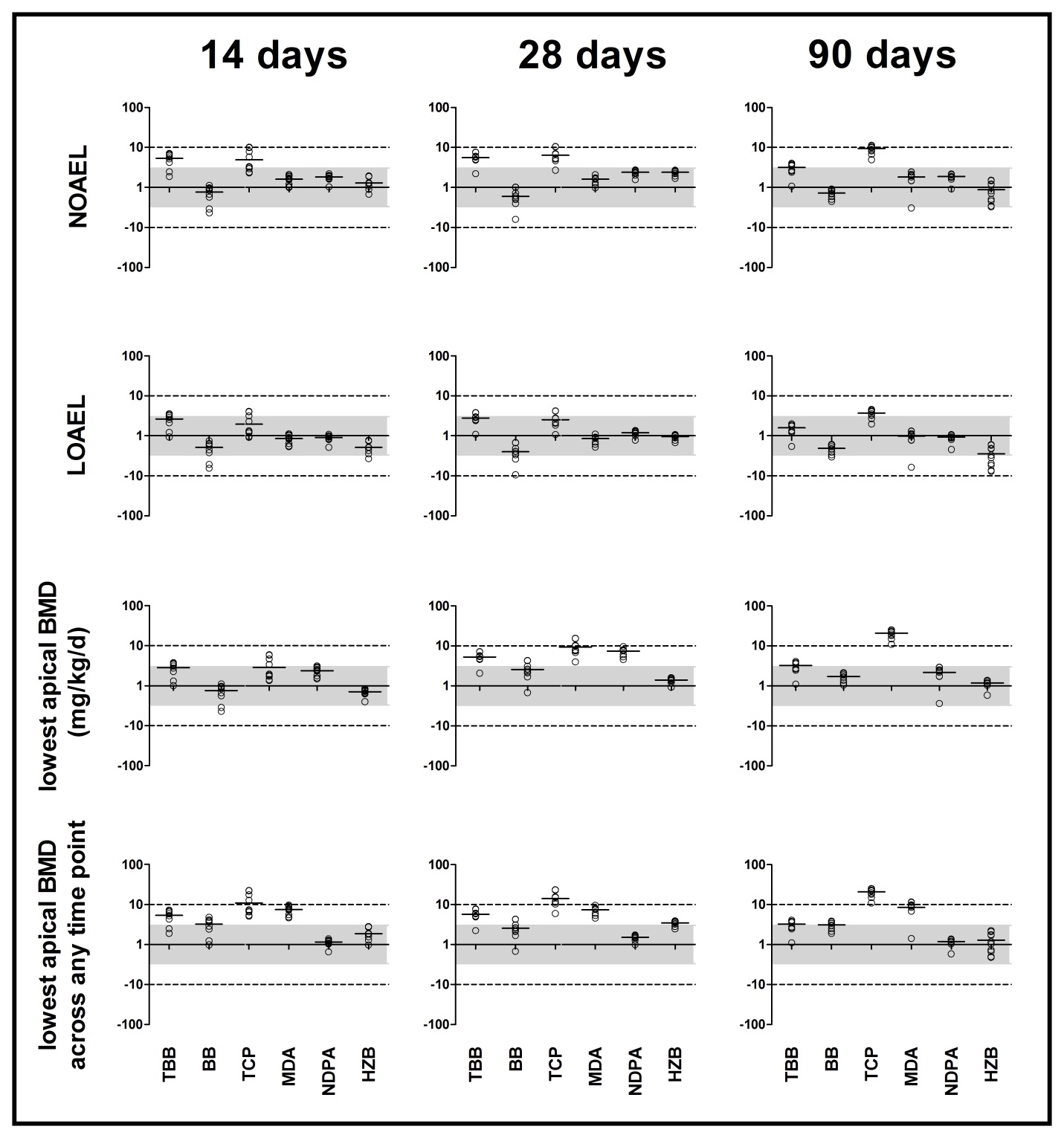


**Figure S7.**


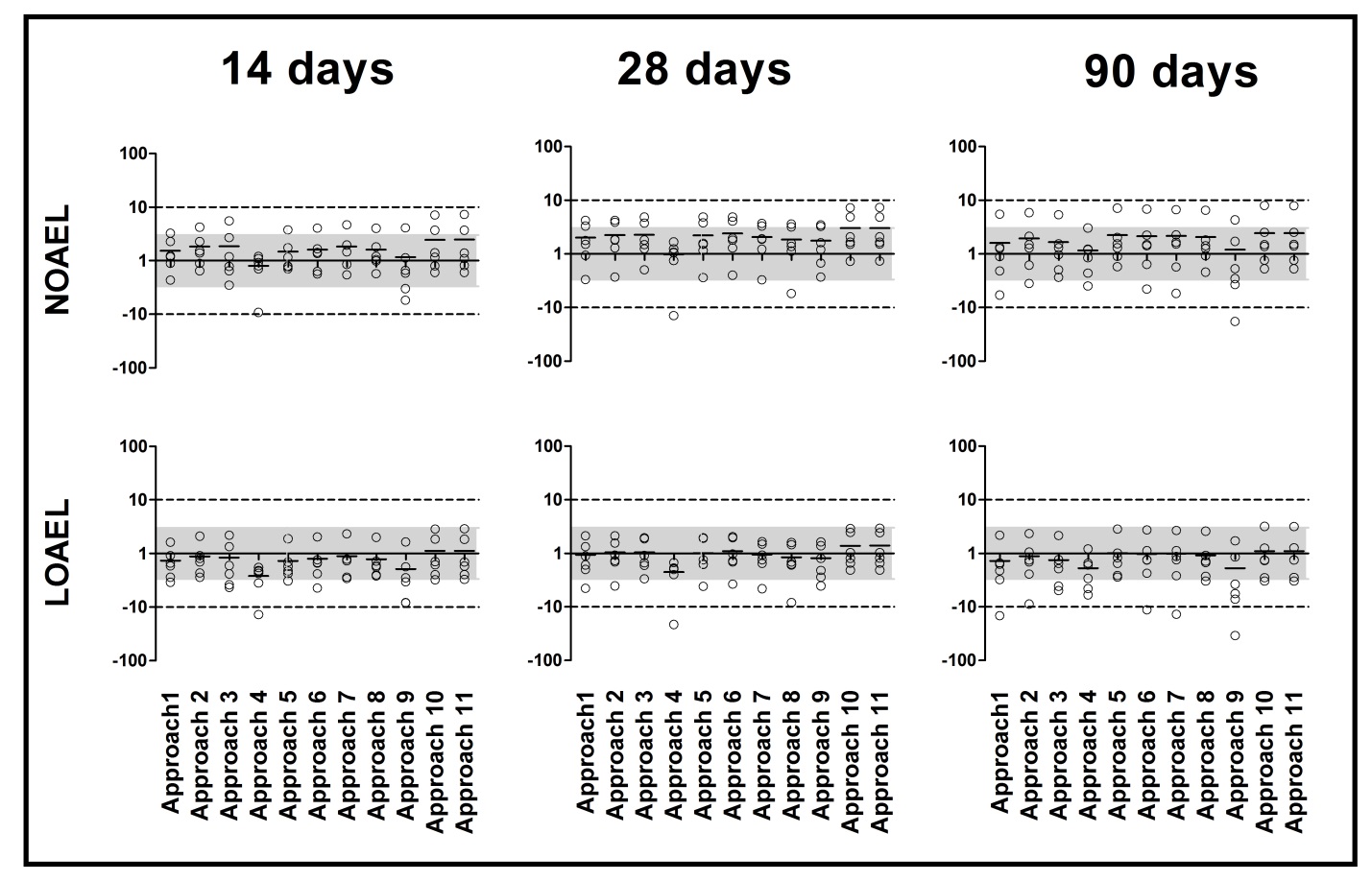


**Figure S8.**

**
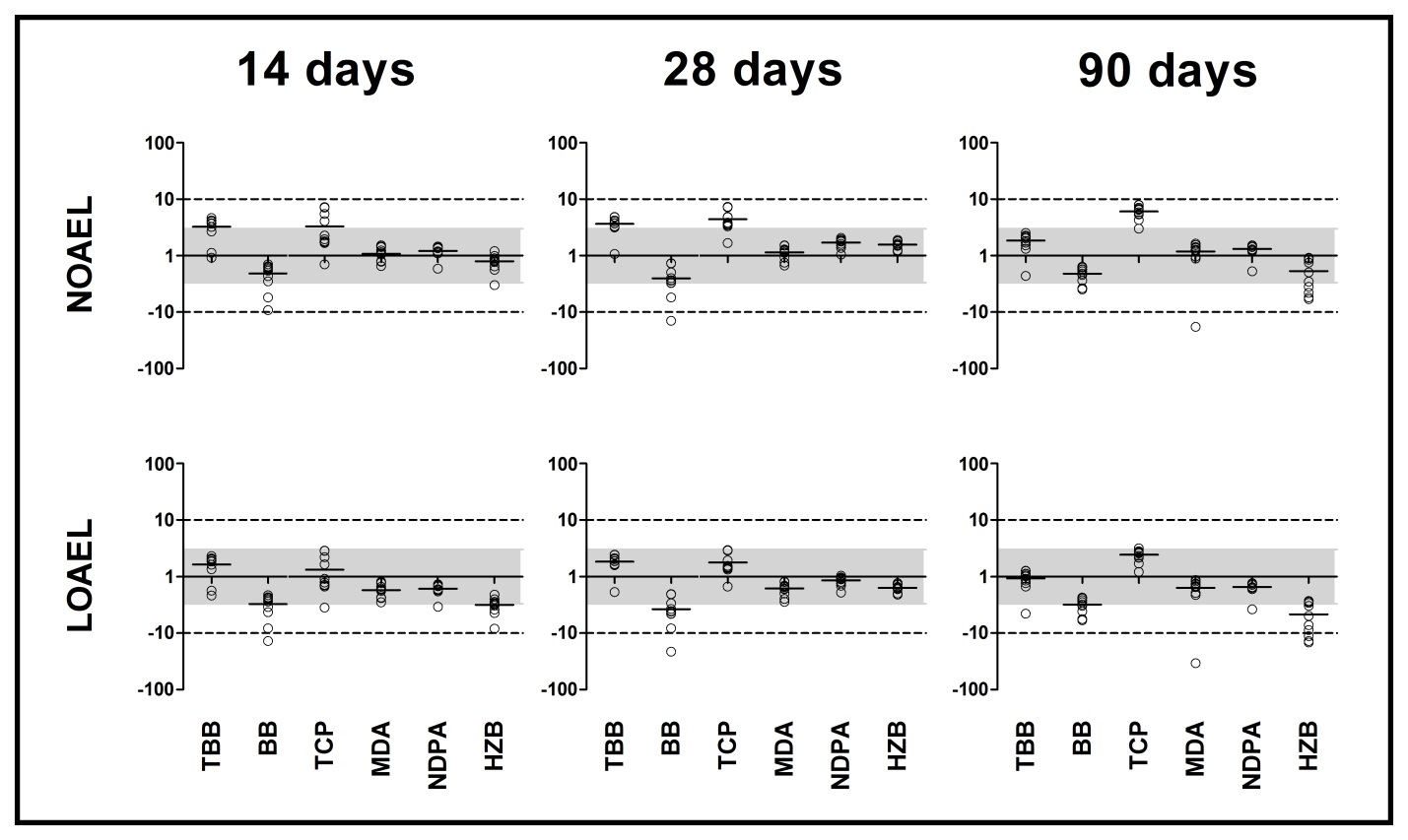
**

**Figure S9.**

**
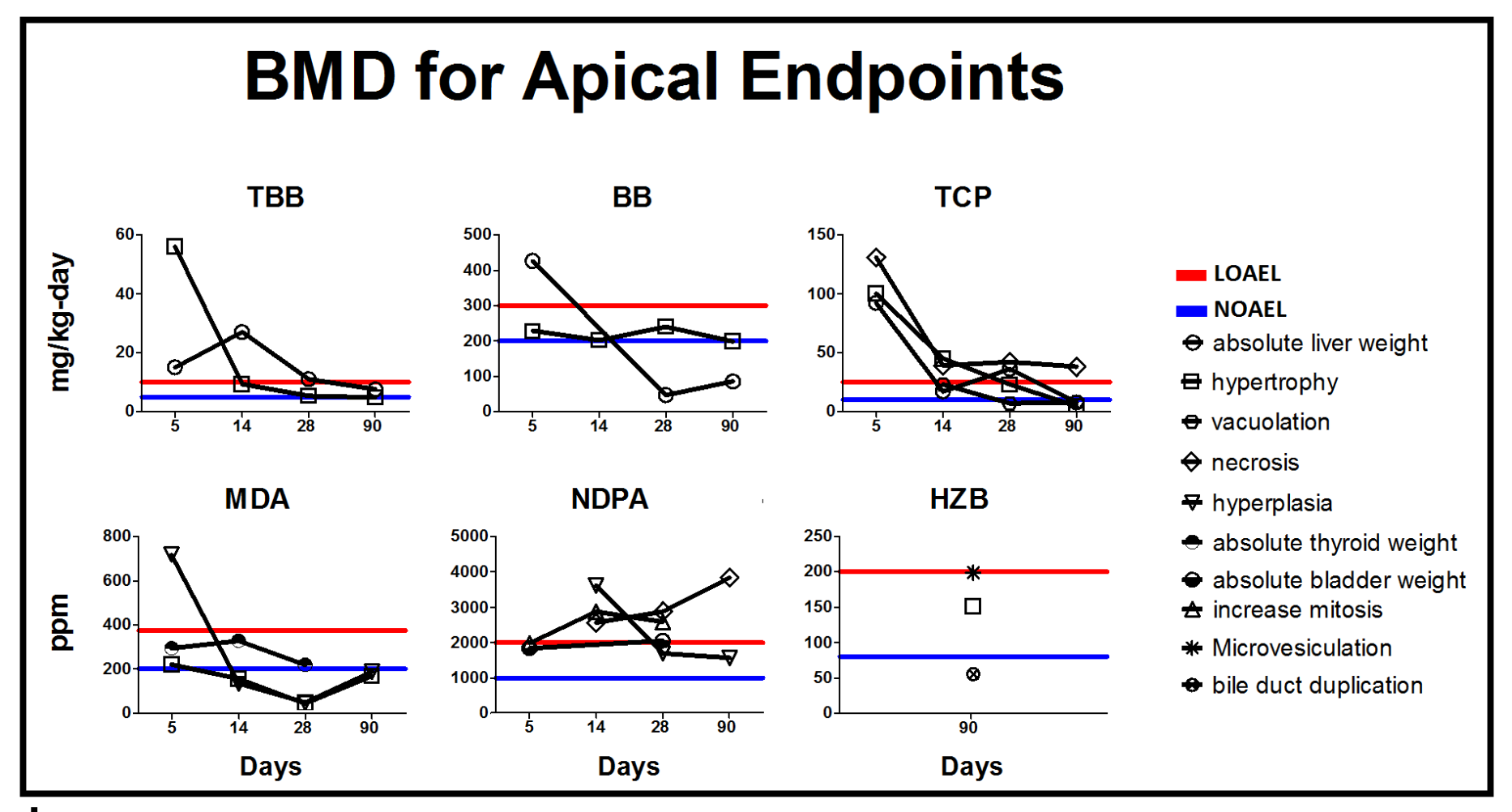
**

**Figure S10.**


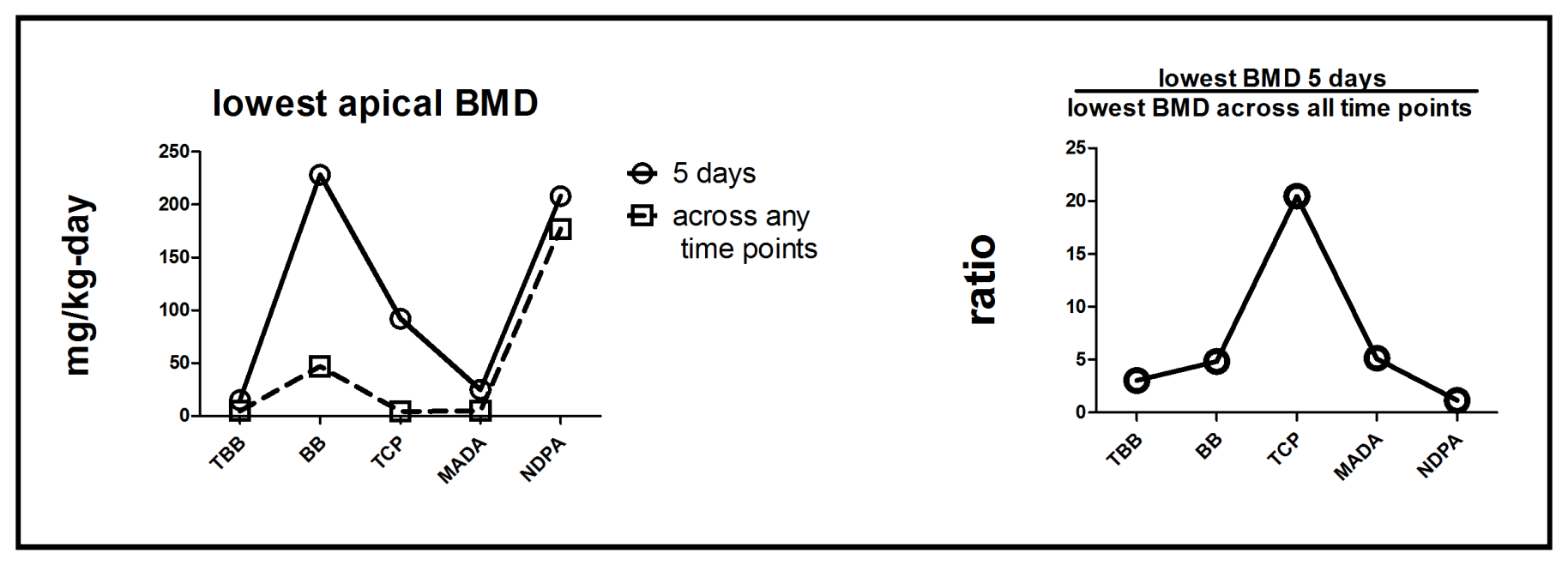


**Figure S11.**

**
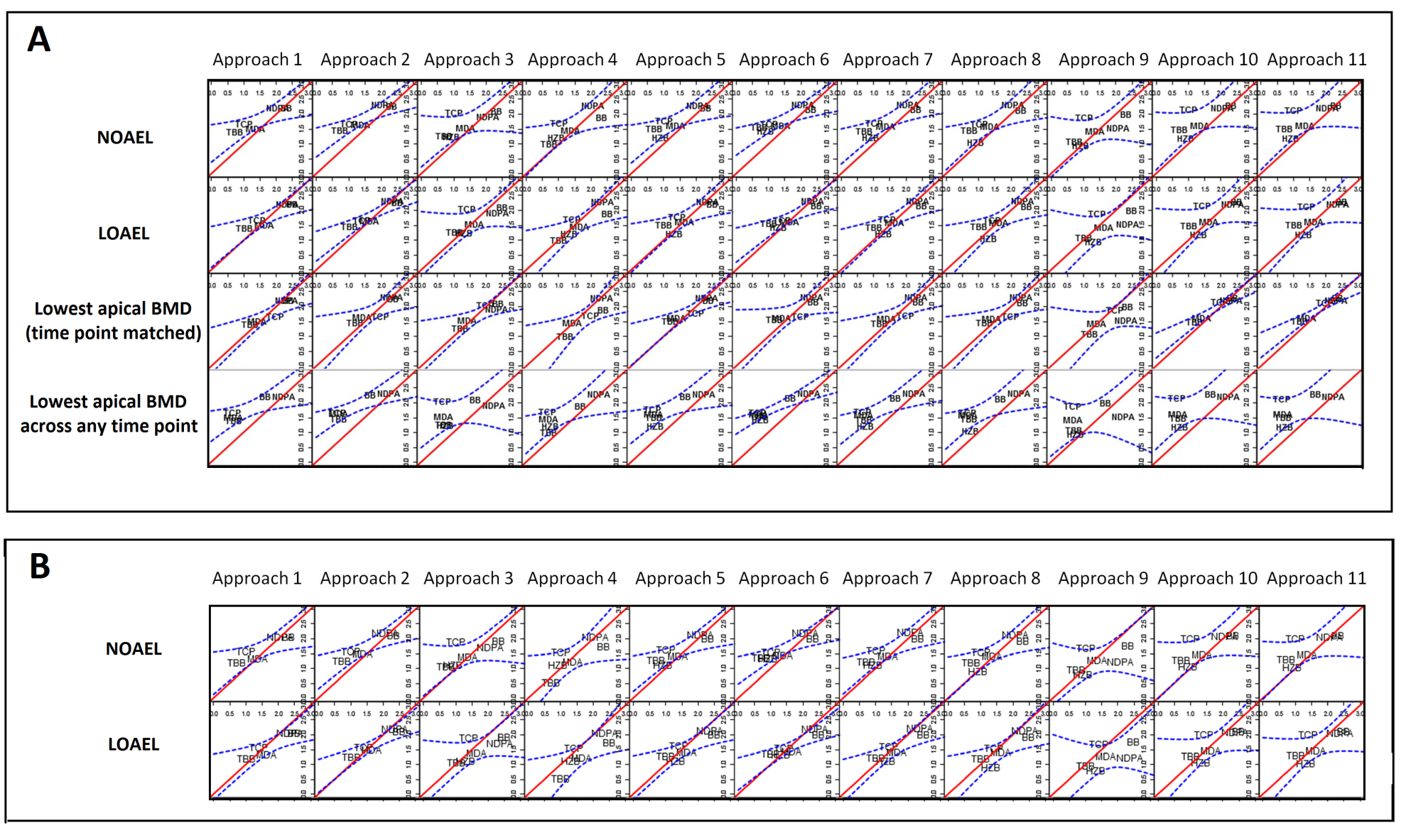
**


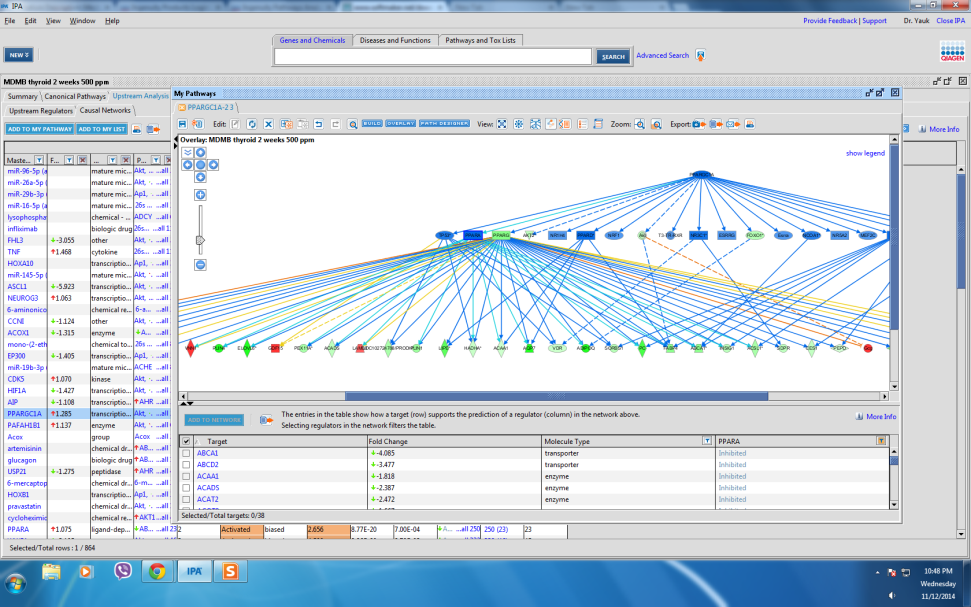
**Figure S12.
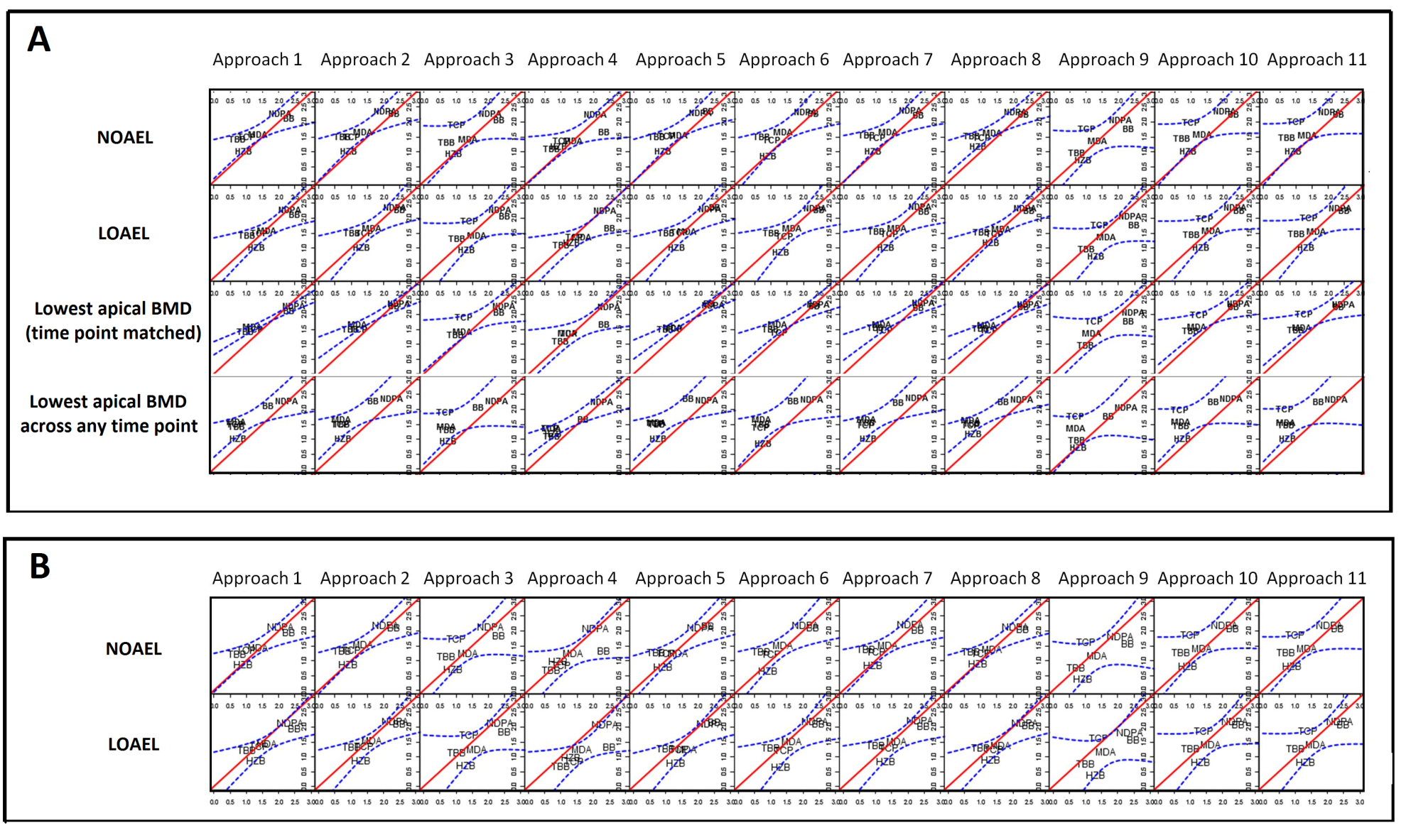
**

**Figure 1.** Overview of study method.

**Figure S13.**

**
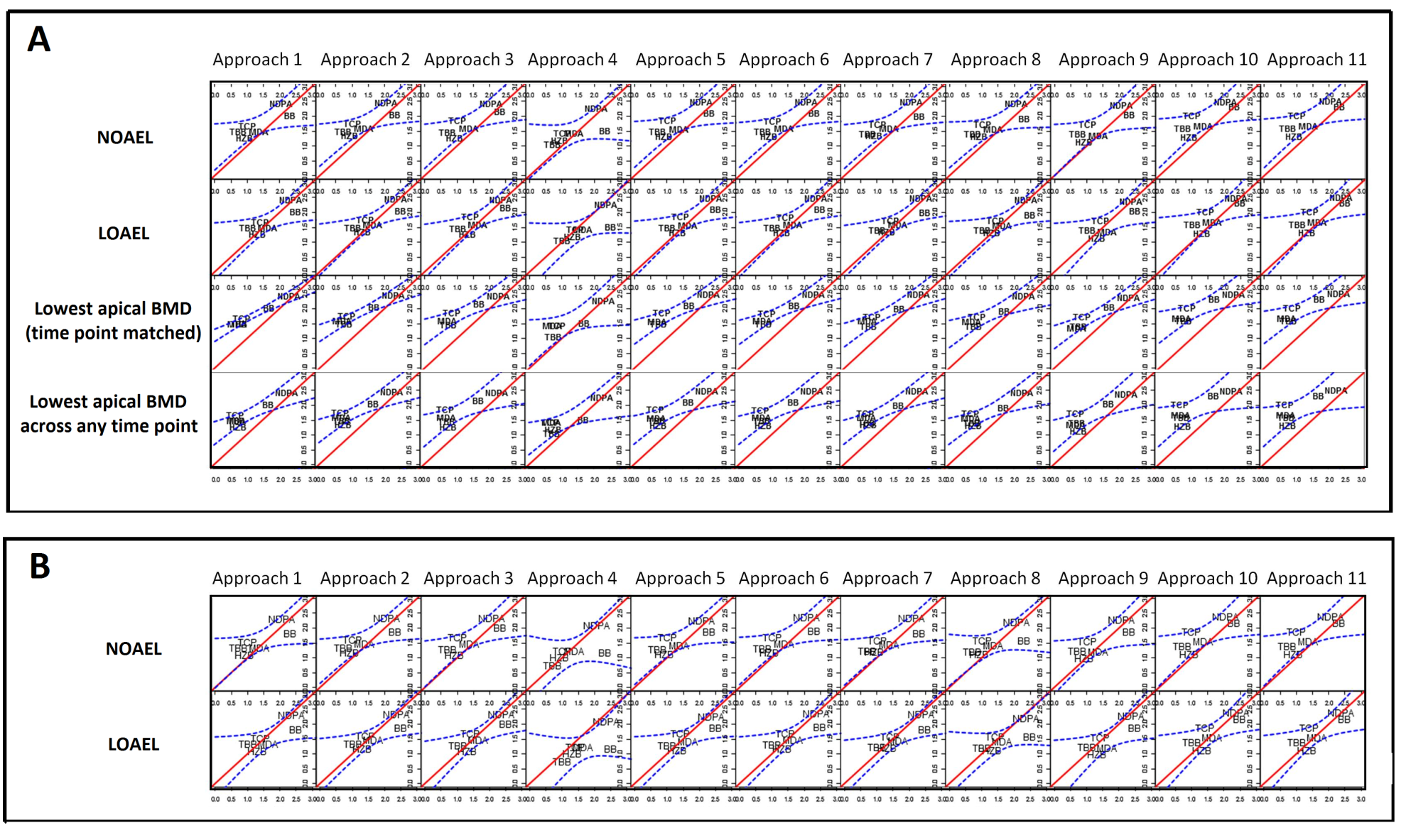
**

**Figure S14.**

**
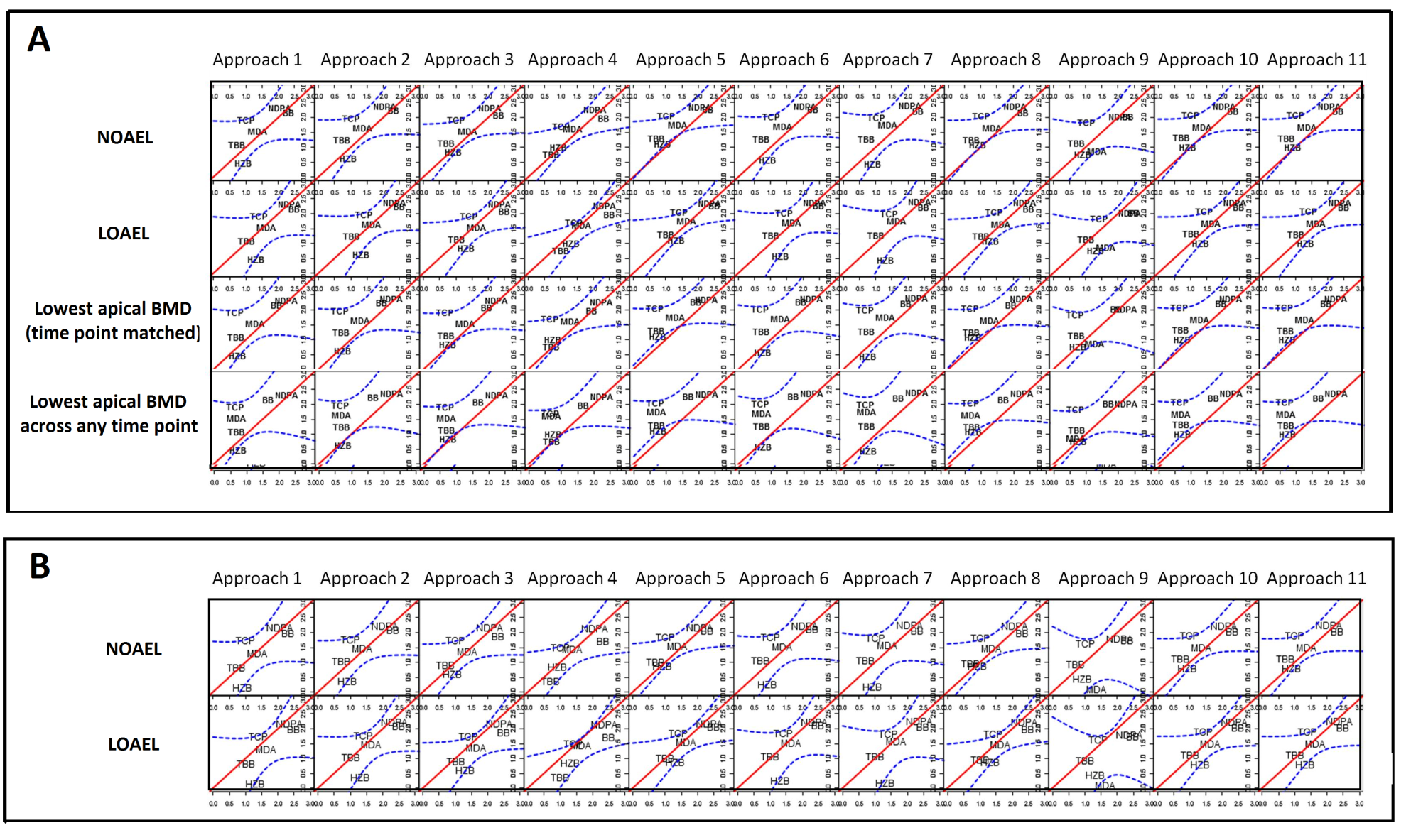
**

**Figure S15.**

**
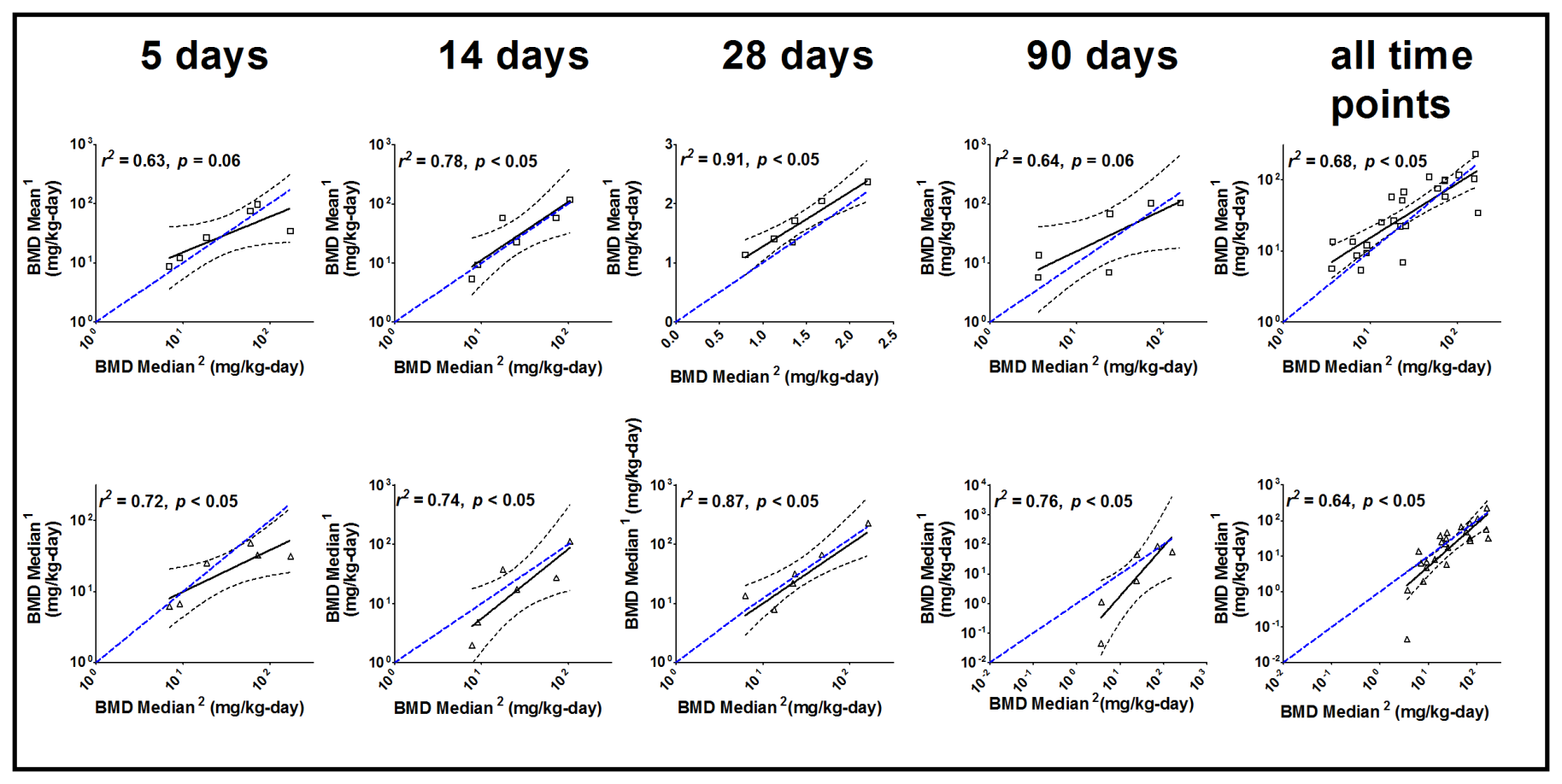
**

**Figure S16.
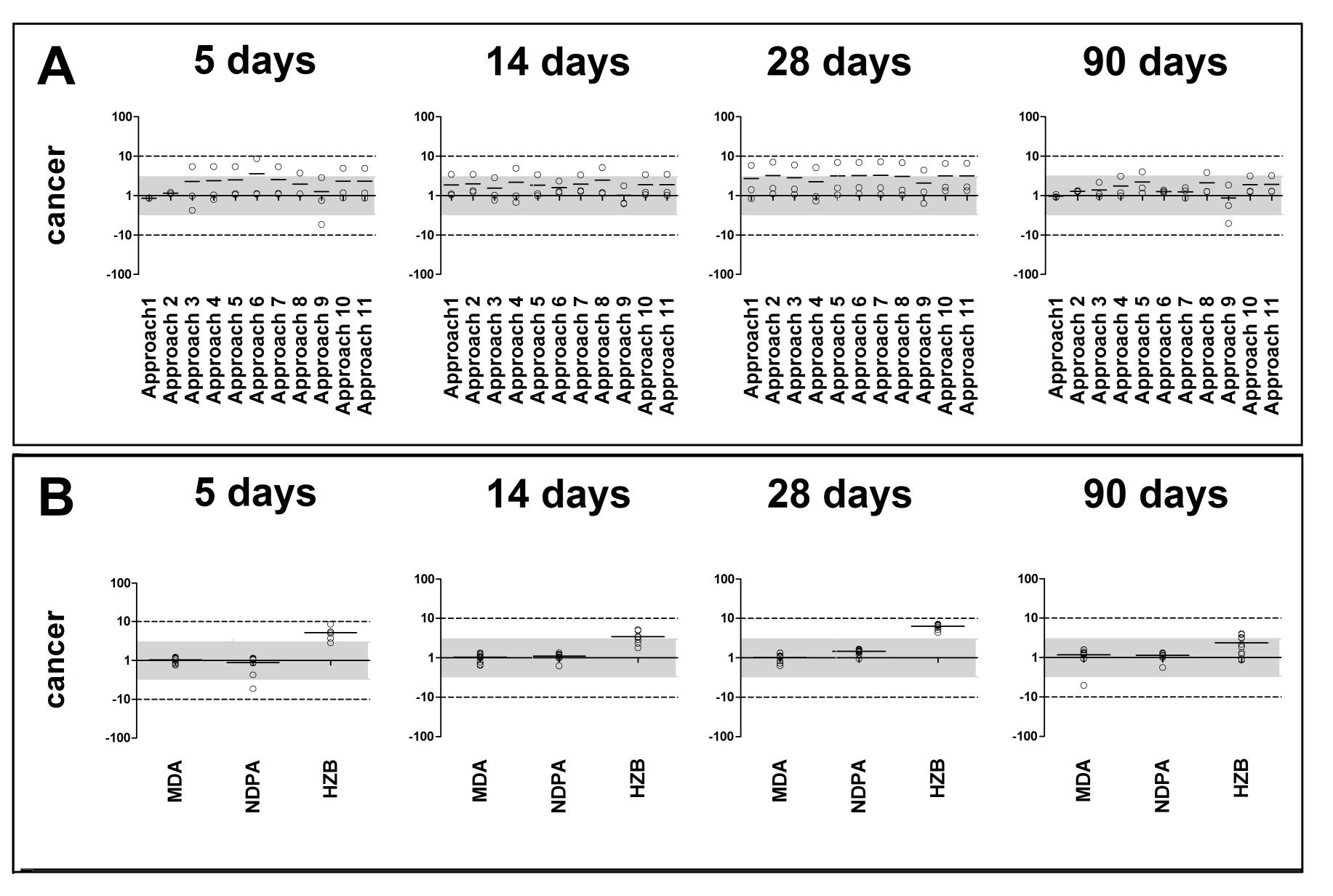
**
