# Supplementary material for: Recommended approaches in the application of toxicogenomics to derive points of departure for chemical risk assessment
Source: Arch Toxicol. 2016 Dec 7;91(5):2045–65. doi: 10.1007/s00204-016-1886-5 (PMC5399047; doi:10.1007/s00204-016-1886-5)
Supplement: Supplementary file 2 — Supplementary material 2 (DOCX 11596 kb) [file 204_2016_1886_MOESM2_ESM.docx]

**Recommended approaches in the application of toxicogenomics to derive points of departure for chemical risk assessment**

Reza Farmahin^1^, Andrew Williams^1^, Byron Kuo^1^, Nikolai L. Chepelev^1^, Russell S. Thomas^2^, Tara S. Barton-Maclaren^3^, Ivan H. Curran^4^, Andy Nong^1^ , Mike G. Wade, and Carole L. Yauk^1*^

^1^Health Canada, Environmental Health Science and Research Bureau, Ottawa, Ontario, K1A 0K9, Canada

^2^National Center for Computational Toxicology, United States Environmental Protection Agency, Research Triangle Park, North Carolina.

**^3^**Existing Substances Risk Assessment Bureau, Healthy Environments and Consumer Safety Branch, Health Canada, Ottawa, Ontario, K1A 0K9, Canada

**^4^**Toxicology Research Division, Health Products and Food Branch, Health Canada, Ottawa, Ontario, K1A 0K9, Canada

^*^ To whom correspondence should be addressed at Health Canada, Environmental Health Science and Research Bureau, Mechanistic Studies Division, 50 Colombine Driveway, Tunney's Pasture, Ottawa, ON K1A 0K9, Canada. Fax: (613) 941-8530. E-mail: [carole.yauk@canada.ca](mailto:carole.yauk@canada.ca)

**Disclaimer:** The views expressed in this paper are those of the authors and do not necessarily reflect the views or policies of the U.S. Environmental Protection Agency. Mention of trade names or commercial products does not constitute endorsement or recommendation for use.

Keywords: transcriptomics, point of departure, chemical, risk assessment, toxicogenomics, microarray, NOAEL, LOAEL, BMD, and BMDL

**ABSTRACT** (250 words)

There is increasing interest in the use of quantitative transcriptomic data to determine benchmark doses (BMD) and estimate a point of departure (POD) for human health risk assessment. Although studies have shown that transcriptional PODs correlate with those derived from apical endpoint changes, there is no consensus on the process used to derive a transcriptional POD. Specifically, the subsets of informative genes that produce BMDs that best approximate the doses at which adverse apical effects occur have not been defined. To determine the best way to select predictive groups of genes, we used published microarray data from dose-response studies on six chemicals in rats exposed orally for 5, 14, 28, and 90 days. We evaluated eight approaches for selecting genes for POD derivation and three previously proposed approaches (the lowest pathway BMD, and the mean and median BMD of all genes). The relationship between transcriptional BMDs derived using these 11 approaches and PODs derived from apical data that might be used in chemical risk assessment was examined. Transcriptional BMD values for all 11 approaches were remarkably aligned with corresponding apical PODs, with the vast majority of toxicogenomics PODs being within 10-fold of those derived from apical endpoints. We identified at least four approaches that produce BMDs that are effective estimates of apical PODs across multiple sampling time points. Our results support that a variety of approaches can be used to derive reproducible transcriptional PODs that are consistent with PODs produced from traditional methods for chemical risk assessment.

**Introduction**

Animal-based toxicity testing is expensive, time-consuming, and requires large numbers of animals. For example, the National Toxicology Program (NTP) estimates that a rodent cancer bioassay requires 860 animals, $2–$4 million, and five years to plan, conduct, and evaluate (NIEHS 1996). As a result, the NTP has generally only conducted an average of 12 cancer bioassays/year since this program was launched in the 1970s. Further, due to the need for animal-based toxicity data for hazard identification and dose-response analysis for many chemicals requiring risk assessment, the US Environmental Protection Agency’s (EPA) Integrated Risk Information System (IRIS) has only evaluated 570 chemicals since the EPA created the IRIS program (1985) up until March, 2016 (<http://www.epa.gov/iris>). In Canada, an important challenge is the requirement to assess the potential for risk to human health of a large number of existing chemicals in a short timeframe. Specifically, the Government of Canada, under the Chemicals Management Plan launched in 2006, has a commitment to address 4,300 exisiting substances identified as priorities by 2020; many of these substances have a paucity of traditional toxicology data (Barton-Maclaren et al. 2016). Traditional whole animal testing is not feasible for the regulatory testing of all chemicals requiring evaluation, hence there has been an increase in the need for, and application of, non-traditional tools and approaches to support decision-making as the programme progresses.

In light of the tens of thousands of chemicals in commerce, and the thousands of new chemicals being developed annually, an urgent need to shift from these conventional toxicology tests towards higher throughput mechanistic and quantitative approaches has been identified (Committee on Toxicity Testing and Assessment of Environmental Agents 2007; Council of Canadian Academies; Firestone et al. 2010; NRC 2009; NRC 2010). Multiple alternative approaches have been proposed to improve and expedite chemical testing, including the application of toxicogenomics, cell-based assays, high-throughput testing, and computational modeling. In particular, toxicogenomics has been identified as important in the next generation of risk science (Chepelev et al. 2015; Cote et al. 2016; Guyton et al. 2009; Krewski et al. 2014).

Alteration in mRNA expression following chemical exposure is one of the earliest quantifiable effects in a toxicological response. Genomics technologies, such as DNA microarrays and RNA-sequencing (RNA-seq), measure global transcriptional changes in a tissue or cell type following chemical exposure. Abundant evidence indicates that changes in mRNA levels occur during chemical toxicity, and that characterizing these changes can provide meaningful information for toxicological assessment (Thomas et al. 2001; Thomas et al. 2013a). Analyzing mRNA expression changes in cells or tissues following toxicant exposure offers a new dimension to hazard and mode of action (MOA) identification, assessment of human and animal variability in response to chemicals, and estimation of the doses at which adverse non-cancer and cancer effects occur (Hester et al. 2015; Jackson et al. 2014; Labib et al. 2015; Moffat et al. 2015; Thomas et al. 2007; Thomas et al. 2011; Thomas et al. 2012; Webster et al. 2015a). The use of transcriptomic data has been suggested for informing the MOA of a chemical as part of a weight of evidence in risk assessment (Bourdon-Lacombe et al. 2015; NRC 2007), and recently it has been proposed that quantitative transcriptomic data may be used to determine benchmark dose (BMD) to estimate a chemical’s point of departure (POD) (Moffat et al. 2015; Thomas et al. 2013b; Webster et al. 2015a).

Numerous studies have applied BMD modeling to analyze dose-response relationships for global gene expression data. These studies have found that transcriptional PODs are in agreement with PODs derived using apical endpoints (e.g., histology, organ weight, cancer, etc.) (Auerbach et al. 2015; Black et al. 2014; Bourdon et al. 2013; Dong et al. 2015; Thomas et al. 2007; Thomas et al. 2013a; Thomas et al. 2013b; Webster et al. 2015a). For example, Thomas *et al.* (2013) reported a high degree of correlation between transcriptional BMD values for the ‘most sensitive pathway’ (i.e., the lowest median pathway BMD) and BMD values for apical endpoints in rodent dose-response experiments for six chemicals over a variety of exposure durations (5, 14, 28, and 90 day exposures). A study on mice exposed to the carcinogen furan for 3 weeks reported that the overall mean/median pathway BMD was consistent with hepatocellular adenoma and carcinoma BMDs from both DNA microarrays data and RNA-seq data (Webster et al. 2015a). It has also been demonstrated that BMDs for pathways associated with key events in a chemical’s MOA are good predictors of the doses at which associated adverse apical effects occur (Bhat et al. 2013; Chepelev et al. 2015; Hester et al. 2015; Webster et al. 2015a). Overall, there is agreement that transcriptional BMDs representing defined groups of genes provide a potentially effective approach for establishing a POD, but there is no consensus on how the genes that are used should be selected (e.g., lowest pathway BMD, BMD from genes in a pathway that is associated with a key event in the chemical’s MOA, overall median/mean transcriptional BMD, etc.) and what the repercussions of different agencies applying different approaches might be on the ultimate PODs used in risk assessment. Thus, a thorough analysis of different methods for estimating a transcriptional BMD and recommendations for best practices are required before application of transcriptional PODs in human health risk assessment can be realized.

The primary purpose of this study was to expand on the previous work described above by systematically exploring different approaches to selecting genes from transcriptional data to derive a POD, and comparing these to PODs derived from apical endpoints within the same rodent models. To do this, we leveraged published Affymetrix microarray data on well-designed dose-response studies in rats with matching (derived from the same rats) apical data. BMDExpress (Yang et al. 2007) was used to derive BMD and lower confidence limit benchmark dose (BMDL) values for gene expression. Eleven approaches to select groups of genes and molecular pathways that are relevant to a chemical’s toxicity were evaluated for derivation of a transcriptional BMD that is representative of the chemical response (Table 1). Three of these approaches are based on methods proposed previously, including BMD mean or median of all responsive pathways, and the lowest pathway BMD (Thomas et al. 2013a; Webster et al. 2015a). These BMD(L) values were compared to the BMD(L)s for conventional toxicology endpoints from the same animals, and to cancer bioassay results published elsewhere. Because MOA-determination is time-consuming, the present work focuses on approaches that can be applied for relatively rapid transcriptional POD derivation without MOA knowledge; this provides an additional advantage for the application of the approach for the assessment of substances with limited toxicity data. The overarching goal of this work is to advance the utility and application of transcriptional BMDs for use in human health risk assessment.

**Materials and Methods**

The data used in this study were publicly available and were selected for use because they contain both transcriptomic and matching apical endpoints across a variety of time points and across a dose-range spanning the no observed effects levels and the maximum tolerated doses. Thus, they provide an ideal dataset for this analysis. Details of the original experiment are described below.

Summary of Experimental Model and Previously Published Work

Details of the study design, animal exposures, necropsy, histology, serum clinical chemistry, blood concentration of chemicals, and microarray methods were reported previously (D. E. Dodd et al., 2012, 2012; Dodd et al., 2013, 2012, 2012, 2012; R. R. Thomas et al., 2013) (Figure 1). In brief, (1) male Sprague-Dawley rats were administered 1,2,4-tribromobenzene (TBB; CAS No. 615-54-3) at 0, 2.5, 5, 10, 25, or 75 mg/kg per day (mkd) by oral gavage; (2) male Fischer 344 (F344) rats were administered bromobenzene (BB; CAS No. 108- 86-1) at 0, 25, 100, 200, 300, or 400 mkd by oral gavage; (3) male Sprague-Dawley rats were administered 2,3,4,6-tetrachlorophenol (TCP; CAS No. 58-90-2) at 0, 10, 25, 50, 100, or 200 mkd by oral gavage; (4) female F344 rats were exposed to 4,4'-methylenebis(N,N'-dimethyl)aniline (CAS number 101-61-1; MDA) in feed at six doses (0, 50, 200, 375, 500, or 750 ppm); (5) female F344 rats were exposed to N-Nitrosodiphenylamine (NDPA; CAS No. 86-30-6) by dietary feed at 0, 250, 1000, 2000, or 4000 ppm; and (6) female F344 rats were exposed to hydrazobenzene (HZB; CAS No. 122-66-7) by dietary feed at concentrations of 0, 5, 20, 80, 200, or 300 ppm (Table 2). The selected rodent strain, sex, and route of exposure were those that showed the critical effect in the previous IRIS assessment for these chemicals. Feed or oral gavage concentrations were selected based on the doses used in the IRIS reviews. Rats were administered or fed the chemicals for 5, 14, 28, or 90 days. Two hundred and forty five 4- to 5-week old rats were used for each chemical (n=10 per group). Clinical signs of toxicity, body weights, and food consumption of animals were checked daily. Necropsies were conducted at scheduled time points. Following gross examination for abnormalities, the target organs (target organs were selected based on previous studies of the test article) were removed, weighed, and prepared for histopathological assessment and gene expression microarray measurements.

For RNA analysis, target tissues were either flash frozen (TBB, TCP, and HZB) or preserved in RNA*later* (BB, MDA, and NDPA) at the time of necropsy. RNA isolated from the primary target tissues of six rats per dose per time point was analyzed using Affymetrix microarrays. Because all of the chemicals selected in this study had published toxicological data, target tissues were known in advance. Target tissues were liver (TBB, BB, TCP and HZB), thyroid (MDA), and bladder (NDPA). DNA microarray hybridization was performed for 16 h on HT Rat230+PM microarrays. The complete microarray datasets were downloaded from the Gene Expression Omnibus (GEO) at the NCBI (Accession No. GSE45892).

Gene expression data were normalized using Robust Multi-array Average (RMA) (Irizarry et al. 2003) and log2 transformed.

Benchmark Dose Calculation

Because we are modeling both apical and transcriptional changes, to avoid confusion, we refer to a BMD derived from an apical endpoint as a BMD_a_, and from a transcriptional endpoint as a BMD_t_ throughout the manuscript. When both BMD and BMDL are discussed, BMD(L) is used. Unless otherwise stated, BMD(L)_a_ values in the current paper refer to non-cancer apical endpoints.

*BMD Analysis of Apical Effects (BMD_a_).* BMD(L)_a_ values were modeled for apical endpoint measurements (described in Dodd *et al.*, 2012a-d, Dodd *et al.*, 2013a-b) using the US EPA’s Benchmark Dose Software (BMDS, version 2.60) (Davis et al. 2011) with BMDS Wizard (ICF international, version 1.1). The dose-dependent histological changes including hypertrophy, hyperplasia, necrosis, and vacuolation were fit as dichotomous data, and organ weight increases were fit as continuous data. All models specified in the BMD modeling guidelines (U.S. EPA 2012) were run for the appropriate data type (dichotomous data: Gamma, Dichotomous-Hill, Logistic, LogLogistic, Probit, LogProbit, Weibull, and Multistage; continuous data: Exponential 4, Exponential 5, Hill, Power, Polynomial, and Linear). The best-fitting model was selected based on adequacy of the fit of the model to the data using automated rules in BMDS wizard (U.S. EPA 2012). No manual interpretation of results was performed; BMD(L)_a_ values were selected based on the program recommendation as described previously (Wignall et al. 2014). The BMDS wizard categorized fitted models into Viable, Questionable or Unusable. Only Viable model outputs were used in this study. If no model was Viable the highest dose was removed and the models were re-run. If no model was Viable after removing the highest doses and only three doses remained (including control), the dose-response data set was reported as having failed BMD modeling. For BMD_a_ values that were higher than the highest dose, the BMD(L)_a_ was not considered and “failed BMD modeling” was recorded.

The BMD_a_ calculations for cancer are described in detail in a previous study (Thomas et al. 2013a). Briefly, cancer BMD_a_ values for thyroid carcinoma/adenoma incidences, bladder carcinoma, and liver carcinomas for MDA, NDPA, and HZB, respectively, were calculated using BMDS software.

*BMD Analysis of Transcriptional Changes (BMD_t_).* BMDExpress was used for dose-response modeling and BMD_t_ estimation for each gene (Yang et al. 2007). A statistical test was used as a pre-filter to identify genes that were significantly altered in at least one dose group relative to concurrent control rats (statistical test applied for each approach is described in Table 1, and described in more detail below). Subsequently, a best fit model (Hill, Power, Linear, Polynomial 2°, or Polynomial 3°) was identified for each gene based on: (1) a nested chi-square test cutoff of 0.05 to choose between linear and polynomial models; (2) the least complexity based on the Akaike Information Criterion (AIC) for the Linear, Polynomial, Hill, and Power models; and (3) a goodness of fit test p-value > 0.1. Other parameters included: power restricted to ≥ 1, maximum iterations of 250 (the convergence criteria for the model fitting), confidence interval of 0.95, and benchmark response (BMR, the number of standard deviations at which the BMD is defined) set to 1.349 (Yang et al. 2007). The Hill model was flagged if the ‘*k*’ parameter was < 1/3 of the lowest positive dose (Black et al. 2012). In such cases, the next best model with a goodness of fit test p-value > 0.05 was selected. In the case where no model had a *p*-value > 0.05, probes that fitted Hill models were considered and the lowest BMD_t_ value (only BMD_t_ derived from Hill models excluding flagged models) was multiplied by 0.5 for use in subsequent analyses. Using the built-in Defined Category Analysis feature, probes with BMD_t_s were mapped to Ingenuity Pathway Analysis (IPA) canonical pathways (downloaded on April 24, 2014). Promiscuous probes (probes annotated with more than one gene), as well as probes with BMD_t_s higher than the highest dose and goodness of fit test p-value < 0.1, were removed.

Identification of Differentially Expressed Genes.

Differentially expressed genes (those with mRNA levels that were significantly increased or decreased following chemical exposure relative to concurrent controls) were identified using Microarray Analysis of Variance (MAANOVA). This analysis was conducted in R (R Core Team 2015) using the MAANOVA library (Wu et al. 2009)*.* The Fs statistic (Cui et al. 2005) was used to test for treatment effects. The p-values were estimated by the permutation method with residual shuffling followed by the false discovery rate (FDR) adjustment (Benjamini and Hochberg 2007). The fold change estimates were determined using least-square means (Goodnight JH 1978; Searle et al. 2012; 1980)*.*

Identification of Perturbed Pathways and Upstream Regulators

*Ingenuity Pathway Analysis (IPA).* Gene expression data were analyzed using QIAGEN’s Ingenuity® Pathway Analysis (IPA®,QIAGEN Redwood City, [www.qiagen.com/ingenuity](http://www.qiagen.com/ingenuity)) to identify significant enrichment of genes in specific molecular pathways and to predict activated upstream regulators. IPA Core Analysis with a gene expression threshold of fold change ≥ ±1.5 and FDR *p* ≤ 0.05 was run and enriched canonical pathways that were statistically significant (*p* ≤0.05) were selected. IPA calculates the p-value using the right-tailed Fisher’s Exact Test. In this method, the p-value for a given pathway is calculated by considering the number of differentially expressed genes (FC ≥ ±1.5 and FDR *p* ≤ 0.05) that participate in that pathway and the total number of genes that are known to be associated with that pathway.

*BMDExpress Data Viewer.*  Pathway enrichment analysis using the BMDExpress Data Viewer tools is described in detail elsewhere (Kuo et al. 2015). Briefly, the datasets derived from BMDExpress were used in this analysis. The Affymetrix probe sets were first converted into unique genes (based on NCBI Entrez Gene ID). The output files (BMD_t_ analyzed and IPA mapped files) were uploaded to the BMDExpress Data Viewer Functional Enrichment Analysis tool. This tool performs enrichment analysis using a Fisher’s exact test. The Fisher’s exact test is identical to conventional pathway analysis (e.g., IPA), except it only applies the analysis to genes that passed the BMD_t_ filtering criteria and have a BMD_t_. Thus, it explores pathway enrichment for genes that show a dose-response and significantly increased in at least one dose group relative to controls. A list of significant pathways (*p <* 0.05) for each dataset is obtained. Only pathways with four or more genes with BMD_t_ values were considered.

Transcriptomic-based Approaches to Predict POD

Eight novel approaches were explored to select and group genes for BMD_t_ analysis (Approaches 1-8). The specific details of each approach are described in Table 1 along with a very brief rationale for why this approach was considered. The mean of the gene BMD_t_s within the groups defined by these approaches was calculated. The correlation between the BMD_t_s derived for each approach and the BMD_a_ values (as well as no and lowest observable adverse effects levels: NOAEL and LOAEL, respectively) was computed. We also used three approaches (Approaches 9-11) that have been used previously (Thomas et al. 2013a; Webster et al. 2015b) to derive BMD_t_s (details again provided in Table 1). The BMD_t_s derived from each of the 11 approaches are presented and analyzed as potential PODs for application in risk assessment.

We note that previous approaches to derived transcriptional BMDs have applied different statistical filtering prior to BMD modeling, ranging from no statistical filter, to a conservative filter of FDR *p* ≤ 0.05 and an unadjusted *p* ≤ 0.05. We previously showed that it is important to pre-filter global transcriptional data prior to BMD_t_ modeling to reduce noise (Webster et al. 2015a). Thus, we applied two analytical methods for pre-filtering data – MAANOVA FDR *p* ≤ 0.05 and ANOVA unadjusted *p* ≤ 0.05 (Table 1) to include both a conservative and liberal filter, respectively, in the present study. In approaches 1, 2, 4, 5, 6, 7, and 8, microarray data were normalized using RMA, a MAANOVA was performed, and genes with FDR *p* values ≤ 0.05 and fold changes ≥ 1.5 were imported into BMDExpress. In approaches 3, 9, 10, and 11, RMA normalized data were directly imported into BMDExpress and analyzed by ANOVA (retaining genes with *p <* 0.05). The subsequent BMDExpress analysis was similar for all approaches following these pre-filtering steps (Table 1). For our analysis, a pathway was only assigned a BMD_t_ if it had a minimum of four genes with BMDs within that pathway. The approaches are also described in detail in Supplementary methods. The mean BMD for each gene with a BMD within a pathway was used to represent the BMD_t_ for that pathway. BMDs derived from each approach represent the mean gene expression BMD for the following groups of genes:

- Approach 1 - The 20 significantly enriched pathways with the lowest BMD_t_s.
- Approach 2 - The 20 most statistically significantly enriched pathways.
- Approach 3 - The 20 lowest pathway BMD_t_s.
- Approach 4 - The 20 genes with the largest fold changes relative to controls**.**
- Approach 5 - Genes with BMD_t_s within the 25^th^-75^th^ percentile.
- Approach 6 – The 20 pathways with the highest number of shared genes.
- Approach 7 - The 20 genes that contribute to the greatest number of enriched pathways.
- Approach 8 – The BMDs of genes that are regulated by the 20 most significant upstream regulators.
- Approach 9 - The significantly enriched pathway with the lowest BMD_t_ (i.e., most sensitive pathway).
- Approach 10 – The mean of gene BMDs across all pathways.
- Approach 11 - The median gene BMD across all pathways.

Estimating Distributions for each Approach

Distributions for the mean or median (Approach 11 only) BMD_t_ for the 11 approaches were estimated using the bootstrap (Efron B 1993). BMD_t_ estimates for genes or for genes within pathways were randomly selected with replacement. For those approaches that employed pathway information, the mean gene BMD was used to estimate the pathway BMD_t_. To estimate the BMD_t_ for each approach, the mean across pathways or the mean across genes was used for each bootstrap. A total of 2000 bootstraps were used to approximate the distribution for each of the 11 approaches. The mean values were used as the transcriptional POD for each approach (except Approach 11) in the subsequent analyses in the manuscript because the mean values had a lower variance than the median values (Figure 2; Figure S1).

Correlation Analysis

Pearson correlations were estimated using the R statistical package. In this analysis BMD and BMDL values in ppm were converted to milligrams per kilogram-day using strain-specific subchronic food intake factors (Female F344 rats is 0.113 and male F344 is 0.1) calculated based on recommended biological values from the EPA (1988). Linear associations between the NOAEL, LOAEL, and the apical endpoint were visualized using scatterplots. The one to one line and the 95% confidence curves were also displayed for each approach. These results were visualized using scatterplots.

Likelihood Ratio Test

A likelihood test was used to test each approach to determine whether it was statistically significantly different from the 1:1 ideal line (the [null](https://en.wikipedia.org/wiki/Null_hypothesis) model). The likelihood ratio statistic is the difference in the likelihood function under the alternative hypothesis and the likelihood function under the null hypothesis; this difference is then multiplied by 2. The likelihood statistic is distributed as a Chi-square distribution with the degrees of freedom equal to the difference in dimensionality of the parameter space under the alternative and null hypothesis. In this analysis the degrees of freedom is 2.The null model was rejected if the p-value was less than 0.05.

Three Criteria for Assessing Approaches

We applied three criteria to assess the effectiveness of the approaches in identifying a relevant POD: (1) the mean ratio of the BMD(L)_t_ derived from an approach should be less than 3-fold the apical POD; (2) significance of the Pearson's correlation coefficient (*p*- value) should be less than 0.05; and (3) the significance of the likelihood ratio test (*p*- value) in deviating from the 1:1 slope should be greater than 0.05.

**RESULTS AND DISCUSSION**

Apical Data

BMD(L)_a_ values were calculated for changes in target organ weight and histology (Table 3). For each chemical, the lowest BMD_a_ value for these apical endpoints within a time point, and across all time points was determined (Figure S2; Figure S3). Because we are not using these data for human health risk assessment, no additional considerations were made (e.g., the relevance of apical endpoint to human health, reversibility of effect, the impact of the allometric exponent, etc.). Generally, the BMD_a_ values decreased over time. The lowest BMD_a_ values were observed at 90 days for TBB, TCP, NDPA, and HZB, and 28 days for BB and MDA. The lowest BMD_a_s across all time points by our calculations were: 4.9 mkd for TBB (hepatocyte hypertrophy), 1567 ppm for NDPA (diffuse transitional epithelial hyperplasia), 55 ppm for HZB (bile duct duplication), 47 mkd for BB (increase in absolute liver weight), 4.5 mkd for TCP (hepatocyte hypertrophy), and 43 ppm for MDA (follicular cell hypertrophy). The lowest BMD_a_s across all time points for TBB, NDPA, and HZB were similar to the NOAEL values (5 mkd, 1000 ppm, and 80 ppm, respectively) reported in previous studies (Dodd et al. 2012b; Dodd et al. 2012d; Dodd et al. 2013a). However, the BMD_a_ values for BB, TCP, and MDA were approximately 4-, 2-, and 4-fold lower than NOAEL values (200 mkd, 10 mkd, and 200 ppm, respectively) (Dodd et al. 2012a; Dodd et al. 2012c; Dodd et al. 2013b). We note that no histopathological changes were observed for HZB at the 5, 14, and 28 day time points (Dodd et al. 2012b); thus, BMD_a_ values were not calculated. We used BMD_a_s as apical PODs, but also considered previously published NOAEL and LOAEL values. Although BMD_a_s offer several advantages over NOAELs or LOAELs (e.g., less dependence on dose spacing, statistical criteria, and making full use of the information on the shape of the dose-response curve (EFSA 2009)), BMD_a_ analyses also have some limitations. For example, severity of the effect observed in apical endpoints (e.g., histopathology observations in Dodd et al papers were generally ranked 1, 2, 3, 4, or 5 representing a minimal, light/mild, moderate, moderately severe, or severe/high incidence), which is an important factor for NOAEL and LOAEL, is not considered in BMD_a_ derivation. Thus, we decided to compare BMD_t_s derived from transcriptional data to NOAEL, LOAEL, as well as the lowest BMD_a_ at the matched time point and the lowest BMD_a_ overall of all of the apical data.

Genomics Data

*General Characteristics.* The number of significantly differentially expressed genes (FC ≥ 1.5 and FDR *p* ≤ 0.05) at each time point generally increased in a dose-dependent manner, but did not appear to be time-dependent (Table 4). Lists of differentially expressed genes and the BMD(L)_t_ values in response to TBB, BB, TCP, MDA, NDPA, and HZB for all time points are shown in Table S1-S24.

*Visual inspection of the distributions of transcriptional and apical BMDs.* Eight approaches to selecting gene and molecular pathway BMD_t_ s for derivation of a transcriptional POD were applied as well as three previously proposed approaches (approaches summarized in Table 1). The mean BMD_t_s of genes and pathways for each approach were calculated (all gene BMDs available in Tables S1-S24; data available upon request for each approach in 43 supplementary tables).

Distributions of BMD_t_s for each approach were visualized by box and whisker plots (Figure 2 and Figure S4). Figure 2 includes horizontal colored lines to indicate the candidate POD values for apical endpoints that would be considered in a risk assessment, including the cancer BMD_a_ (when available), NOAEL and LOAEL values for apical endpoints measured in the rodents in this study, and the lowest BMD_a_s for these animals. Visual inspection of Figure 2 suggests a high degree of overlap in the BMD_t_s and ranges for each of the approaches despite being drawn from, in many cases, very different gene lists. Approach 9 (the significantly enriched pathway with the lowest BMD_t_) generally produces the lowest BMD_t_s, but also appears to have the broadest interquartile ranges, suggesting a higher degree of variability and uncertainty in applying this approach. In contrast, approaches 10 and 11 (mean and median of all pathways) yield BMD_t_s that are somewhat higher (with tighter distributions), but are remarkably similar to the majority of the other approaches, despite representing the entire selection of pathway BMD_t_s rather than the most statistically significant, or lowest BMD_t_s. It was not possible to apply Approach 1 or 2 to HZB at the 5 day time point because there were no pathways that were significantly enriched in IPA, indicating a limitation of this approach. Approach 4, based on the 20 genes with the greatest fold changes, tended to have lower BMD_t_s than the other approaches. We note that coefficient of variation (CV) values (Tables S25-S26) were below 0.2 for all approaches except 9, which indicates relatively low dispersion of data points around the mean BMD_t_ of these approaches. Overall, visual inspection suggests that the majority of the approaches yield comparable BMD_t_ values, within 10-fold of the corresponding BMDa, that are largely consistent with the various PODs derived from apical endpoint analysis. Below we explore the relationship of the BMD_t_s derived in each of the approaches to BMD_a_s in more detail.

Relationship between Transcriptional and Apical Endpoint BMDs

The BMD_t_s derived from each approach were divided by the POD values (NOAEL, LOAEL, lowest BMD_a_ at matched time point, and lowest BMD_a_ overall) for each chemical to explore the relationship between transcriptomic-based PODs derived from each approach to apical PODs (Figure 3, Figures S5- S8). From Figure 3, it is evident that the majority of BMD_t_s fall within 3-fold of the NOAEL and LOAEL (Figure 3-panels A and B). We compared the BMD_t_ at 5 days to the lowest overall BMD_a_ to see how predictive early BMD_t_s would be for later apical effects (Figure 3-panels A and B). It is worth noting that the BMD_a_s generally decline over time (Figure S9), and the BMD_a_ at day 5 is always greater than the lowest BMD_a_ across all times as expected (Figure S10). We find that the BMD_t_s across all time points are somewhat higher than the lowest BMD_a_ from the target tissue across all time points, but nonetheless, are generally still within 10-fold.

The correlation between log-transformed BMD(L)_t_s derived from each of the approaches and the log-transformed apical POD values (including lowest BMD_a_, NOAEL, LOAEL, and the time-point matched lowest BMD_a_s) were also calculated to determine coefficient correlations (*r*) and linear significance (*p-* values) in order to determine the extent of correlation between transcriptional and apical data. Figure S11 shows the 5 day time point for this correlation analysis; the other time points are shown in Figures S12, S13, and S14; Table 5; Tables S27 and S29. The linear relationship between the apical POD values and BMD(L)_t_s for the 11 approaches was tested to assess whether it approached a 1:1 relationship using the likelihood ratio test (Table 5 and Tables S27-S29). Overall, we found strong correlations between BMD(L)_t_ values derived from each approach and apical POD values. The *r* values for all approaches (264 *r* values were derived) were within the range of 0.54 to 0.99 across the data set (67%, 67%, 84%, and 18% of the correlations were statistically significant at 5, 14, 28, and 90 days, respectively). Assessment of the BMD(L)_t_ values derived from the 11 approaches clearly showed that the transcriptional data were closely aligned with LOAELs, followed by NOAELs, and then the time-point matched lowest BMD_a_s. The linear regression models for most of the transcriptional approaches were not significantly different from a 1:1 relationship with the apical data.

We applied three criteria to assess the effectiveness of the approaches in identifying a relevant POD. For simplicity, because of the size of the dataset, we have focussed on presenting the results from the 5 day time point for each chemical in more detail, and place these findings in the context of the other time points (Table 5). The complete results obtained from 14, 28, and 90 day time points are found in the supplementary materials (Tables S27-29).

*BMD(L)_t_s compared to the NOAEL for apical effects.*

On day 5, the maximal differences between BMD_t_ values for each chemical and the NOAEL derived from analysis of apical endpoints were less than 10-fold for 61 of the 64 data sets (11 approaches x 6 chemicals – 2* = 64; * Approach 1 and 2 could be performed for 5 chemicals only); analysis of TCP using Approach 3, 10, and 11 yielded BMD_t_s that were greater than 10-fold the NOAEL (Figure 3-panels A and B). Indeed, the BMD_t_:NOAEL ratios for the majority of the approaches (42 out of 64 data points) were smaller than 3. Analysis of the average of this ratio across all of the chemicals for every approach revealed that the BMD_t_ mean derived using Approach 4 was closest to the NOAEL (2-fold greater than the NOAEL; Figure 3-panel A). In addition, a significant correlation between the BMD_t_ mean values derived using Approach 4 and the NOAEL (*r* = 0.84, *p<* 0.05) was found (Figure S11; Table 5- panel A). Moreover, BMD_t_s derived using Approach 4 plotted against apical NOAEL values was not significantly different from the 1:1 (ideal) line (Table 5- panel A; Figure S11). Based on the three criteria, Approach 4 is thus the most effective at predicting the NOAEL at the 5 day time point, followed by Approach 1, 2, 7, 8, and 9 that meet two criteria each.

The results for the other time points were largely consistent with the results at 5 days. The majority of BMD_t_s were within 10-fold, and generally within 3-fold, of the apical NOAELs (Figures S5-S6). Approaches 10 and 11 were again marginally higher than the other approaches for a few data points and Approaches 4 and 9 were generally closest to the NOAEL. The specific number of data points (e.g., for each chemical and each approach = 66 data points) within 3-fold at 14, 28, and 90 days were 48/66, 45/66, and 47/66, respectively. Average correlation coefficients for all approaches in the 14, 28, and 90 day data sets were 0.85, 0.82, and 0.77, respectively, similar to average *r* values for 5 day time points (*r* = 0.83). Based on our three criteria, Approaches 1, 4, 5, 6, and 7 at 14 days; Approaches 1, 7, and 9 at 28 days, and Approach 4 at 90 days, were effective in predicting NOAELs.

Overall, the BMDL_t_ mean values were closer to the NOAEL than the BMD_t_ values (Tables S27-S29; Figures S7 and S8). All of the approaches in all time points yielded BMDL_t_s within 10-fold of the NOAEL, and the averages of the BMDL_t_/NOAEL ratios for the six chemicals were less than 3 in all approaches. The number of data points within 3-fold of their associated NOAELs in the 5, 14, 28, and 90 day time point data sets were 42/64, 51/66, 42/66, and 49/66, respectively (Figures S7 and S8). BMDL_t_s derived from all approaches were very highly correlated with their apical endpoint NOAELs in 5, 14, 28, and 90 day time point data sets, and average *r* values for all approaches were 0.82, 0.84, 0.80, and 0.76, respectively (Table 5- panel E; Tables S27-S29). The BMDL_t_-derived approaches that met the three criteria were Approaches 1, 4, 5, 6, 7, and 8 for the 5 day data sets; Approaches 1, 2, 5, 6, 7, and 8 for 14 days; Approaches 2, 3, 6, 7, 9, 10, and 11 for 28 days; and Approaches 4, 5, and 8 for 90 days.

*BMD(L)_t_s compared to the LOAEL for apical effects.*

At the 5 day time point, the BMD_t_ values for all of the approaches for all six of the chemicals were within 10-fold of LOAEL values, and predominantly within 3-fold of the LOAELs as well. Only 8 of the 64 data points were outside of the 3-fold range (Figures 3: panels A and B). In general, the average BMD_t_s were remarkably similar to the LOAELs across all of the approaches. The average BMD_t_s for Approaches 4 and 9 were slightly below the LOAEL. Indeed, the BMD_t_ mean values for all data points in approaches 1, 2, 7, and 8 for all chemicals were within 3-fold of the LOAEL (Figure 3). The averages of the BMD_t_/LOAEL ratios for the six chemicals were very close to 1 in all cases, with Approach 9 having the largest divergence from 1 (ratio = 1.0; CV = 0.318). In general, all approaches except 3, 9, 10, and 11 were significantly correlated with the LOAEL (Pearson’s correlation). The largest and smallest correlation coefficients were found for Approach 2 (*r* = 0.97; *p* value =0.005) and Approach 9 (*r* = 0.65; *p* value =0.16), respectively (Table 5- panel B; Figure S11). The likelihood ratio test results showed transcriptional-to-LOAEL linear regression models for all approaches were not significantly different from 1:1, except for Approach 2. Based on the three criteria, BMD_t_ values derived from Approaches 1, 4, 5, 6, 7, and 8 most accurately predict the LOAEL at the 5 day time point (Table 5- panel B; Table 6- panel B).

The results for the other time points were largely consistent with the results at 5 days (Figures S5 and S6). All of the BMD_t_s were within 10-fold of the LOAELs, and generally within 3-fold of the LOAELs. The number of data points within 3-fold of LOAELs for the 14, 28 and 90 day data sets were 55/66, 60/66, and 50/66, respectively. Indeed, at 14, 28, and 90 days the means of the BMD_t_:LOAEL ratios for all approaches were very close to 1. The maximum and minimum correlation coefficients between the approaches and their matched LOAELs were 0.91 and 0.77 in the 14 day data sets, 0.87 and 0.77 in the 28 day datasets, and 0.88 and 0.70 in the 90 day data sets. The likelihood ratio test results showed transcriptional-to-LOAEL linear regression models for all approaches in the 14, 28, 90 day time points were not significantly different from 1:1. Our results show that seven, ten, and three of the approaches met our three criteria and may be recommended for predicting the LOAEL for the 14, 28, and 90 day time points (Table 5- panel B; Table 6- panel B; Table S27-S29).

For the 5 day time point, the BMDL_t_ values for all approaches were within 10-fold of the LOAEL (except for NDPA, approach 9), and only 10 of the 64 data points were outside of the 3-fold range (Figures S7 and S8). All of the data points from Approach 1, 2, 5, 6, and 7 were within 3-fold of the LOAEL. All approaches were highly correlated with the LOAEL (Table 5- panel F). Similar to the BMD_t_ analysis, the BMDL_t_ values for Approach 2 (*r* = 0.97; *p* value =0.001) and 9 (*r* = 0.55; *p* value =0.25) had the highest and lowest correlation coefficients with apical data. The BMDL_t_ values for Approaches 2, 3, 5, 6, 7, and 8 were closest to the LOAEL (Figures S7 and S8). The likelihood ratio test results showed no significant difference from the 1:1 line for any of the approaches in the 5 day data sets (Table 5- panel F).

Similar results were found in our analysis of BMDL_t_s for the other time points. All approaches (except Approach 4 at 14 days and 28 days, and Approaches 1, 6, 7, and 9 at 90 days) were within 10-fold of the LOAEL, and there were 11/64, 8/66, and 18/66 data points outside the 3-fold range in the 14, 28, and 90 day time points, respectively. The BMDL_t_s values for all approaches and time points were generally highly correlated with the LOAELs. Out of the 11 approaches, BMDL_t_s from seven, nine, and three of the approaches in the 14, 28, and 90 day time point data sets, respectively, were significantly (*p <* 0.05) correlated with their respective LOAELs. The maximum and minimum *r* values were 0.90 and 0.72 at 14 days, 0.87 and 0.69 at 28 days, and 0.87 and 0.57 at 90 days (Tables S27-S29). No significant difference from the 1:1 line (BMD_t_ versus LOAEL) was found, with the exception of Approach 4 (14 days) and Approaches 4 and 9 (90 days). Overall, seven, seven, nine, and two of the approaches in the 5, 14, 28, and 90 day data sets (respectively) met the three criteria, and thus may be recommended for predicting the LOAEL (Table 5- panel F; Table 6- panel F; Tables S27-S29).

*BMD_t_s compared to time-point matched lowest BMD_a_.* The results of current study show that BMD_t_ values derived from transcriptomic data for most of the approaches were remarkably close to the lowest BMD_a_ at 5 days. The average ratios of BMD_t_ -to-BMD_a_ values at the 5 day time point for all approaches were less than 1.5, with Approach 9 having the largest divergence from 1 (Table 5- panel C; Figure 3). BMD_t_s for all approaches on day 5, with one exception (Approach 9, NDPA), were within 3-fold of the lowest BMD_a_ at the 5 day time point (Figure 3). These results suggest that at the 5 day time point similar doses are required to trigger both transcriptional and apical responses. The BMD_t_s for all approaches, except Approach 9, were significantly correlated with the lowest BMD_a_s at 5 days. The maximum and minimum correlation coefficients were 0.98 (*p <* 0.01) for Approaches 10 and 11, and 0.81 (*p =* 0.1) for Approach 9 (Table 5- panel C; Figure 3). There were no significant deviations from the 1:1 line for transcriptional-to-apical comparisons, except for Approaches 10 and 11 (Table 5- panel C). Thus, all approaches except 9, 10, and 11 meet the criteria and are effective predictors of time-point matched BMD_a_s (Table 6 – panel C).

Analysis of the other time points was largely consistent with the results at 5 days (Figures S5-S6). The overwhelming majority of BMD_t_s at 14 days were within 10-fold and generally within 3-fold of the lowest time-matched BMD_a_, with no greater than a 2.6-fold mean BMD_t_ : BMD_a_ ratio for any approach. For the 28 and 90 day time points, the mean BMD_t_ : BMD_a_ ratios for all 11 approaches were greater than 3, with the exception of Approach 4. BMD_t_s for 51 of the 55 and 55 of the 66 approaches were within 10-fold of BMD_a_s at the 28 and 90 day time points, respectively. It should be noted that all BMD_t_ : BMD_a_ ratios that yielded a value greater than 10 were derived from TCP. These results indicate (for the current chemicals) that gene alterations and apical effects occurred at similar doses on day 5; however, at later time points, time-matched apical responses generally occurred at somewhat lower doses than transcriptional responses (Tables S27-S29). However, we note that the vast majority of the BMD_t_s even at the 90-day time point were within 10-fold of apical PODs, suggesting their utility even at 90-days. Similar to 5 days, all approaches except 9, 10, and 11 meet the criteria for day 14 data sets. However, only Approach 4 met all three criteria at the 28 and 90 day time points (Table 6 – panel C).

Thomas *et al.* (2013) proposed the use of the lowest pathway BMD_t_ for prediction of BMD_a_s. Using this approach, a strong correlation (*r* > 0.90) was found between BMD_t_s and the lowest time-point matched BMD_a_ values for adverse apical endpoints at 5, 14, 28, and 90 day time points for these same six chemicals (Thomas et al. 2013a). In the current study, two main modifications were made to this method (Approach 9). The modifications were to: (1) apply the analysis only to ANOVA pre-filtered genes (*p* < 0.05 in at least one dose group); and (2) to remove pathways that were not significantly enriched following BMD_t_ analysis (Fisher’s exact test; *p* ≤0.05). We directly compared our BMD_t_s to those published in Thomas *et al.* (2013) (Figure S15) and found that they were highly comparable. Specifically, our results were similar to those of Thomas *et al.* (2013) at the 5, 14, and 28 day time points (*r* = 0.81, 0.81, 0.97, respectively), but not at 90 days (*r* = 0.61). These results suggest that addition of a Fisher’s exact test to this Approach did not improve relationships with BMD_a_s. Indeed, the linear relationship between BMD_t_s derived from the lowest transcriptional pathway and its time-point matched apical endpoint decreased due to this additional filtering. Because the lowest pathway BMD_t_ may in certain cases be based on only a handful of genes (depending on the minimum number of genes required in a pathway for BMD_t_ consideration that is applied), we felt this additional filtering ensured a more robust and responsive set of genes for this application. Thus, despite the weaker correlation, we advise the application of this filter should this approach be applied.

*BMD_t_s compared to the lowest BMD_a_ across all time points.* At the 5 day time point, the ratio of the BMD_t_s to the lowest overall BMD_a_ (across all time points) tended to be high. However, the majority of the BMD_t_s were within 10-fold of the lowest BMD_a_s at the 5 day time point (Figures 3); of the 64 data points, only five were below 1. Seven ratios were greater than 10, but these were spread across approaches and were all for the chemical TCP (Figure 3-panel B). The correlations between the mean BMD_t_s for Approaches 1, 2, 4, 5, 6, 7, and 8 and the lowest overall BMD_a_s were significant (*p <* 0.05), with *r* > 0.86 (Figure S11). The results of likelihood ratio test showed that at the 5 day time point there were no significant differences between the 1:1 line and transcriptional-to-apical lines for Approaches 3, 4, and 9 (Table 5- panel D). However, no approach met all three of our selection criteria at the 5 day time point (Table 5- panel D; Table 6- panel D). Thus, BMD_t_s are the least effective in predicting the lowest BMD_a_ relative to all of the other apical PODs assessed on day 5.

Analyses of the other time points were similar to the above. Unsurprisingly, the ratios tended to be larger than 1 for BMD_t_s relative to the lowest BMD_a_, although the BMD_t_s across all approaches were predominantly within 10-fold of the BMD_a_. The maximum and minimum correlation coefficients (*r* value) were 0.94 and 0.71 for 14 days, 0.94 and 0.84 for 28 days, and 0.76 and 0.63 for 90 days (Tables S27-S29). There was no significant difference between a 1:1 line and the transcriptional-to-apical lines for Approach 9 (14 days), Approach 4 (28 days), and Approaches 1, 3, 4, 7, and 9 (90 days). However, only Approach 4 (28 days) met all three of our criteria for prediction of the lowest BMD_a_ across all time points (Table 5- panel D; Table 6- panel D; Tables S27-S29).

Overall, we found that while the majority of the approaches were effective predictors of apical PODs at the 5, 14, and 28 day time point, only three approaches (Approaches 4, 5, and 8) met our three criteria at the 90 day time point (Table 6). Approaches 9, 10, and 11 only met the three criteria at the 28 day time point to estimate NOAEL and LOAEL. Approach 9 tended to have larger variation than the other approaches, and Approaches 10 and 11 (mean and median overall pathway BMD_t_s, respectively) tended to overestimate POD as expected. Across the entire dataset, the BMD_t_s were best at predicting the LOAEL and the lowest time-point matched BMD_a_, whereas BMDL_t_s were relatively equal in being effective predictors of LOAEL and NOAEL.

*Overall comparison between transcriptional BMD(L)_t_s and apical PODs.* We estimated the potential ability of the approaches to predict apical POD at each time point based on the sum of the criteria met by BMD_t-_ or BMDL_t_-derived for each approach against apical PODs (Table 7). These results suggest that Approach 7 followed by 1 were the best predictors at 5, 14, and 28 days. At 90 days, however, Approach 4 was the best method to predict apical PODs.

*Transcriptional BMD_t_s compared to the tumor responses.* Tumor responses in thyroid, bladder, and liver, respectively, were reported in rodents following MDA, NDPA, and HZB exposure (NTP 1978; NTP 1979a; NTP 1979b). Incidences of tumor development were analyzed in a previous study to obtain cancer BMD_a_ values (Thomas et al. 2013a). We found that BMD_t_ values for all of the approaches at 5, 14, and 28 days (Figure S16-panel A) were within 10-fold of the tumor response. BMD_t_ values derived from the 11 approaches for MDA and NDPA were within 3-fold at these time points (Figure S16-panel B). The average ratios of BMD_t_ -to-cancer BMD_a_ values at the 90 day time point for all approaches were less than 3. While more carcinogenic chemicals are required to calculate a correlation coefficient, based on the results derived from three chemicals in the current study the transcriptomics-derived BMD_t_ values at 90 days were slightly closer to cancer BMD_a_ values than other time points.

Statistical filtering

BMDExpress is equipped with a built-in tool for filtering a dataset and selecting the probe set (Yang et al. 2007). This tool uses a one-way ANOVA together with a FDR correction for multiple comparisons (Benjamini and Hochberg 2007), and the user is allowed to choose between FDR *p*-value and *p*-value. A previous study from our laboratory investigated the effects of using statistically filtered data on gene and pathway BMD_t_s, and more specifically compared FDR *p*-value with ANOVA unadjusted *p*-value (Webster et al. 2015a). The study showed that pre-filtering data in BMDExpress significantly reduced the mean gene and pathway BMD_t_s. Moreover, transcriptional data that were subjected to more stringent filtering produced BMD_t_s that were closer to apical PODs. These results are not surprising since stringent filtering reduces false positives and ensures that only genes that truly respond to the treatment are considered. However, more stringent filtering methods also reduce the number of discoveries and increase the probability of not retaining a sufficient number of genes for BMD_t_ modeling. For these reasons, Webster *et al.* (2015) recommended that at least an ANOVA *p ≤* 0.05 filter, which is less stringent than an FDR *p*-value filter, is applied to the data prior to modeling in BMDExpress.

Our approaches also applied different statistical tests (MAANOVAFDR *p* ≤0.05 versus ANOVA *p* <0.05) for analyzing and pre-filtering data. Significant genes were selected based on: (1) fold change and corrected *p-* value cut off ( FC ≤ 1.5; FDR *p*-value < 0.05) using MAANOVA in Approaches 1, 2, 4, 5, 6, 7, and 8; and (2) *p*-value ≤ 0.05 using ANOVA in Approaches 3, 9, 10, and 11. Overall, BMD(L)_t_ derived from our MAANOVA-filtered approaches, which applied an FDR correction and fold-change cutoff, were better at predicting apical PODs (86 out 168 approaches met the three criteria; 51%) than those approaches analyzed using ANOVA (15 out 96 approaches met the three criteria; 16%; Table 6). Thus, our results support more stringent pre-filtering of data for deriving BMD_t_s.

Based on the three criteria we described above, the BMD(L)_t_ from approaches that applied a more stringent MAANOVA FDR *p*-value ≤ 0.05 and fold change ≥ 1.5 for gene expression changes were most effective in predicting NOAEL and LOAEL at the 5 day time point. Moreover, all of the MAANOVA-analyzed approaches, as well as Approach 3 (the 20 lowest pathway BMD_t_s) met the three criteria for predicting the most sensitive apical endpoint observed on day 5. However, no approach met the three criteria when analyzed against the lowest BMD_a_ endpoint across all time points at 5 days.

**Conclusions**

We leveraged published Affymetrix DNA microarray data on well-designed time-series and dose-response experiments in rats to evaluate 11 approaches to deriving a BMD_t_ from groups of genes. We assessed the relationship between BMD_t_s derived using these 11 approaches to PODs derived from apical data that might be used in a human health risk assessment. To evaluate the effectiveness of the approaches in predicting apical PODs we used three criteria: (1) ratio of BMD_t_ to apical endpoint POD < 3; (2) correlation coefficient *p*- value < 0.05 for BMD_t_ to apical POD; and (3) likelihood ratio test *p*- value > 0.05 for deviation from the 1:1 line for BMD_t_ versus apical POD. We found a very high degree of concordance between all of the approaches for deriving BMD(L) _t_s and apical PODs. Generally, in our opinion, BMD(L) _t_ values derived using the 11 approaches were remarkably aligned with different apical PODs that may be used in human health risk assessment. The vast majority of BMD_t_s across all approaches were within 10-fold of the various BMD_a_s, and were largely within 3-fold as well. In general, across the 5, 14, 28, and 90 day time points, eight, eight, eleven, and three approaches met our three criteria, respectively, and thus qualify as effective estimates of apical PODs.

Consistency in the PODs derived from transcriptional endpoints with those derived from standard toxicity endpoints increases confidence in the use of transcriptional PODs in human health risk assessment. However, the relevance of these specific gene expression perturbations to adverse effects is unclear, since they are not based on an MOA-centric approach. The approaches described assume that significant perturbations in gene expression in general may lead to an adverse outcome. Early thought in this field presumed that gene expression changes would occur at lower doses than adverse apical effects and thus would be overly conservative. In contrast, we demonstrate that mean transcriptional PODs from the approaches reviewed herein are generally higher than apical PODs, suggesting that this is not the case. Indeed, the mean transcriptional BMDs differ from the corresponding apical endpoints by less than 1.5-fold for matched time points (Table 5, panel C), or are within 10-fold across all time points (Table 5, panel D). These observations suggest that for these chemicals transcriptional changes do not occur at lower doses than apical responses, alleviating concerns that transcriptomics approaches in risk assessment would be overly protective.

Although additional studies using chemicals targeting different types of adverse effects are required to validate our findings, our results suggest that transcriptional response can be used as an efficient alternative approach for POD selection in chemical risk assessment. Transcriptional PODs were furthest from apical PODs at the 90 day time, suggesting that some dampening of transcriptional response may be occurring at later time points, and supporting the use of earlier time points to identify doses that significantly impact transcriptional profiles along a trajectory towards disease. Our results indicate that transcriptionally-derived POD estimation from a short term study are consistently within 10 fold of PODs derived from apical endpoints from longer term studies. We also support previous findings that a more conservative statistical filter yields transcriptional PODs that are more aligned with apical PODs. However, our results suggest that any of the proposed approaches should produce transcriptional PODs that are within 10-fold of the noncancer and cancer BMD_a_ in target tissues. While Approaches 7, 1, and 4 appear to be the best predictors of apical PODs, decisions on which approach is used could be determined based on the formulated risk assessment question, and/or any guidelines established by the regulatory agency undertaking the evaluation if they exist (e.g., (Bourdon-Lacombe et al. 2015)). Overall, our paper suggests that transcriptional analyses produce highly robust BMD_t_ metrics that are strong predictors of apical PODs within an acceptable measure of uncertainty (generally < 10-fold).

The integration of toxicogenomics into human health risk assessment is a relatively new area and no international guidelines on this have been established. Major challenges include selecting the most relevant genes or pathways to patho-physiological effects. Addressing this issue will assist the integration of genomic data into chemical risk assessment. Previous work from our and other laboratories have provided initial examples of how toxicogenomics can be used to inform human health risk assessment, including both hazard identification and dose-response analysis (e.g., (Andersen et al. 2010; Bercu et al. 2010; Bhat et al. 2013; Bourdon-Lacombe et al. 2015; Chepelev et al. 2016; 2015; Chepelev et al. 2016; 2016; Clewell et al. 2011; Cote et al. 2016; Jackson et al. 2014; Moffat et al. 2015; Thomas et al. 2011; Thomas et al. 2012)). It has been noted that toxicogenomics-guided elucidation of MOA information is helpful, as it can guide the selection of MOA-relevant key events and hence, identification of relevant toxicogenomics data that should be used in dose-response analyses (e.g., (Moffat et al. 2015)). However, here we also support the previous contention that toxicogenomics data can be used for dose-response analyses even without detailed MOA information, especially in the case of “non-selective” chemicals (i.e., those interacting with multiple biological targets, and, hence, having the potential to contribute to several MOAs) (Thomas et al. 2013b). Indeed, identifying MOA from toxicogenomics data can be time-consuming, as compounds may operate through several mechanisms perturbing an array of biological effects (Bercu et al. 2010). Therefore, it has been suggested that the lowest dose at which transcriptional perturbations become measurable (e.g., the lowest pathway BMD_t_) should be considered for risk assessment (Thomas et al. 2013b). In this study, we demonstrate that a variety of approaches are amenable to deriving a highly reproducible transcriptional POD, thus not requiring regulatory agencies to select a single approach.

**Acknowledgements**

This research was funded by Health Canada’s Chemicals Management Plan. We thank Drs. Julie Bourdon-Lacombe and Anna Francina Webster for helpful comments on the manuscript.

**FIGURE LEGENDS**

**Figure 1.** Study method overview. White boxes show animal exposures, necropsies, histology and microarray procedures that were conducted in previous studies (D. E. Dodd et al., 2012, 2012; Dodd et al., 2013, 2012, 2012, 2012; R. R. Thomas et al., 2013). Blue boxes represent procedures that were conducted in the current study.

**Figure 2.** Box and whisker plots of the BMD_t_ mean for all approaches for all chemicals at the 5, 14, 28, and 90 day time points. Colored horizontal lines represent the NOAEL (blue line), LOAEL (red line), lowest time-point matched BMD_a_ value for apical endpoint (gray line), the lowest overall BMD_a_ values (i.e., any time) for apical endpoint across all time points (green line), and cancer (black line). The box boundaries and lines represent the interquartile ranges and means, respectively. The whiskers represent 10 and 90 percentiles.

**Figure 3.** BMD(L)_t_s relative to apical PODs for the 5-day time point. Three- and ten-fold ranges from the apical POD are within the shaded area and the dashed horizontal lines, respectively. (A) The BMD_t_s derived from each approach were divided by the corresponding apical POD values for every chemical; (B) data from panel A shown separately for each chemical; (C) the BMDL_t_s derived from each approach were divided by NOAEL and LOAEL; (D) data from panel C shown separately for each chemical.

**SUPPLEMENTARY FIGURE LEGENDS**

**Figure S1.** Box and whisker plots of the BMD_t_ median for the 11 approaches for all chemicals at the 5, 14, 28 and 90 day time points. Colored horizontal lines represent the NOAEL (blue line) and LOAEL (red line). The box boundaries and lines represent the interquartile ranges and medians, respectively. The whiskers represent 10 and 90 percentiles.

**Figure S2.** BMD modeling of continuous data (e.g., organ weight) and dichotomous data (e.g., hypertrophy, hyperplasia, and vacuolation) for the apical endpoint with the lowest BMD_a_ at each time point for TBB, BB and TCP.

**Figure S3.** BMD modeling of continuous data (e.g., organ weight) and dichotomous data (e.g., hypertrophy, hyperplasia, and vacuolation) for the apical endpoint with the lowest BMD_a_ at each time point for MDA, NDPA and HZB.

**Figure S4.** Box and whisker plots of the BMDL_t_ mean for the 11 approaches for all of the chemicals at the 5, 14, 28 and 90 day time points. Colored horizontal lines represent the NOAEL (blue line), LOAEL (red line), lowest time-point matched BMD_a_ value (gray line), the lowest overall BMD_a_ values (i.e., any time) for apical endpoint across all time points (green line), and cancer (black line). The box boundaries and lines represent the interquartile ranges and means, respectively. The whiskers represent 10 and 90 percentiles.

**Figure S5.** BMD_t_s relative to apical PODs for the 14, 28, and 90 day time points. Three-fold and ten-fold ranges from the apical POD are within the shaded areas and dashed horizontal lines, respectively. The BMD_t_s derived from each approach were divided by the POD values for each approach for every chemical.

**Figure S6.** BMD_t_s relative to apical PODs for the 14, 28, and 90 day time points. Data from Figure S5 are shown separately for each chemical.

**Figure S7.** BMDL_t_s relative to NOAEL and LOAEL for 14, 28, and 90 days. Three-fold and ten-fold ranges from the apical POD are within shaded areas and dashed horizontal lines, respectively. The BMD_t_s derived from each approach were divided by the POD values for each approach for every chemical.

**Figure S8.** BMDL_t_s relative to apical PODs for the 14, 28, and 90 day time points. Data from Figure S7 are shown separately for each chemical.

**Figure S9.** BMD_a_ values for apical responses.

Note: pathological changes related to HZB were observed at the 90 day time point only.

**Figure S10.** BMD_a_ at day 5 relative to the lowest apical endpoint across any time point.

**Figure S11.** Correlation between log-transformed BMD_t_ (panel A) and BMDL_t_ (panel B) values derived from the 11 approaches at the 5 day time point and log-transformed POD values. The 1:1 line is indicated as a solid red line, and the 95% confidence interval of the regression line is indicated by dashed blue lines.

**Figure S12.** Correlation between log-transformed BMD_t_ (panel A) and BMDL_t_ (panel B) values derived from the 11 approaches at the 14 day time point and log-transformed POD values. The 1:1 line is indicated by a solid red line, and the 95% confidence interval of the regression line is indicated by dashed blue lines.

**Figure S13.** Correlation between log-transformed BMD_t_ (panel A) and BMDL_t_ (panel B) values derived from the 11 approaches at the 28 day time point and log-transformed POD values. The 1:1 line is indicated by a solid red line, and the 95% confidence interval of the regression line is indicated by dashed blue lines.

**Figure S14.** Correlation between log-transformed BMD_t_ (panel A) and BMDL_t_ (panel B) values derived from the 11 approaches at the 90 day time point and log-transformed POD values. The 1:1 line is indicated by a solid red line, and the 95% confidence interval of the regression line is indicated by dashed blue lines.

**Figure S15.** Correlation between log-transformed mean (top row) and median (bottom row) BMD_t_ values derived from Approach 9 in the current study^1^ at the 5 day, 14 day, 28 day, 90 day, and all time points and log-transformed median BMD_t_ values from Thomas et al (2013)^2^.

**Figure S16.**  ***(A)*** BMD_t_ values derived from the 11 approaches for MDA, NDPA and HZB for 5, 14, 28, and 90 days relative to the cancer BMD_a_ values for incidences of tumor development. Three-fold and ten-fold differences from the apical POD are shown within the shaded areas and dashed horizontal lines, respectively. The BMD_t_s derived from each approach were divided by the BMD_a_ values for every chemical. ***(B)*** Data from panel A are shown separately for each chemical.

**Table 1.** Description of the 11 approaches to derive a transcriptomic POD.

| **Approach** | **Normalization** | **Analysis of Variance** | **Cut off values** | **Pathway mapping** | **Fisher’s exact test (cut off value)** | **Group of gene BMD**_t_**s** | **Rationale** |
| --- | --- | --- | --- | --- | --- | --- | --- |
| **1** | RMA | MAANOVA^1^ | FDR^2^<0.05; FC^3^>1.5 | IPA | *p<*0.05 | 20 pathways with the lowest BMD_t_s | Ensuring analysis of a robust set of the most responsive significantly affected pathways. |
| **2** | RMA | MAANOVA | FDR<0.05; FC>1.5 | IPA | *p<*0.05 | 20 pathways with the lowest *p-*values | As a measure of those pathways that may be toxicologically relevant because they are the most significantly enriched. |
| **3** | RMA | ANOVA^4^ | *p<*0.05 | IPA | *p<*0.05 | 20 pathways with the lowest BMD_t_s | Selecting a robust set of the most responsive genes, regardless of whether the pathway is enriched or not (liberal filter – no FDR). |
| **4** | RMA | MAANOVA | FDR<0.05; FC>1.5 |  |  | 20 genes with the largest fold change | Targeting the most responsive genes that may contribute in a substantive way to the toxicological response. |
| **5** | RMA | MAANOVA | FDR<0.05; FC>1.5 |  |  | genes with BMD_t_ values within the 25^th^ and 75^th^ percentiles | A central measure of response. |
| **6** | RMA | MAANOVA | FDR<0.05; FC>1.5 | IPA | *p<*0.05 | 20 pathways with the highest number of shared genes | Central nodes in pathway interaction networks may be toxicologically relevant because they contain the highest number of shared genes. |
| **7** | RMA | MAANOVA | FDR<0.05; FC>1.5 | IPA | *p<*0.05 | 20 genes that contribute to the greatest number of enriched pathways | Analysis of genes that contribute to the most pathways may encompass genes that are critical to the toxicological response. |
| **8** | RMA | MAANOVA | FDR<0.05; FC>1.5 | IPA | *p<*0.05 | 20 most significant upstream regulators | Upstream regulators may drive key events in the toxicological response and be critical to the mode of action. |
| **9^5^** | RMA | ANOVA | *p<*0.05 | IPA | *p<*0.05 | significantly enriched pathway with the lowest BMD_t_ | Previously suggested (pathway that is perturbed at the lowest dose). |
| **10^6^** | RMA | ANOVA | *p<*0.05 | IPA |  | mean of all pathway BMD_t_s | Previously suggested |
| **11^6^** | RMA | ANOVA | *p<*0.05 | IPA |  | median of all pathway BMD_t_s | Previously suggested |

^1^ MAANOVA (MicroArray ANalysis Of Variance); ^2^FDR (false discovery rate) ^; 3^FC (fold change); ^4^Analysis of variance (ANOVA); **^5^**(Thomas et al. 2013a) with the following modifications: (1) pre- analysis filtration of genes (ANOVA; p< 0.05 in at least one dose group); and (2) to select only pathways that were significantly enriched following BMD_t_ analysis (Fisher’s exact test; p< 0.05) ; **^6^** (Webster et al. 2015a); BMD_a_: BMD derived from an apical endpoint; BMD_t_: BMD derived from a transcriptional endpoint

**Table 2.** Details on chemical treatments, rodent models, and target tissues.

| **chemical** | **CAS number** | **doses** | **rodent model** | **target tissue** |
| --- | --- | --- | --- | --- |
| 1,2,4-Tribromobenzene (TBB) | 615-54-3 | 2.5, 5, 10, 25, and 75 mkd | Male Sprague Dawley rats | Liver |
| Bromobenzene (BB) | 108-86-1 | 25, 100, 200, 300, and 400 mkd | Male F344 rats | Liver |
| 2,3,4,6-Tetrachlorophenol (TCP) | 58-90-2 | 10, 25, 50, 100, and 200 mkd | Male Sprague Dawley rats | Liver |
| 4,4′-Methylenebis (N,N-dimethyl) benzenamine (MDA) | 101-61-1 | 50, 200, 375, 500, and 750 ppm | Female F344 rats | Thyroid |
| N-Nitrosodiphenylamine (NDPA) | 86-30-6 | 250, 1000, 2000, 3000, and 4000 ppm | Female F344 rats | Bladder |
| Hydrazobenzene (HZB) | 122-66-7 | 5, 20, 80, 200, and 300 ppm | Male F344 rats | Liver |

**Table 3.** BMD(L)_a_ values for changes in organ weight and histological effects across different time points, along with NOAEL and LOAEL values.

| **Chemical** | **Apical endpoint** | **5 days** | | **14 days** | | **28 days** | | **90 days** | |  |  |  |
| --- | --- | --- | --- | --- | --- | --- | --- | --- | --- | --- | --- | --- |
|  |  |  |  |  |  |  |  |  |  | **NOAEL*** | **LOAEL*** |  |
| **TBB (mkd)** | absolute liver weight | *15* | (7) | 27 | (21) | 11 | (5.9) | 7.6 | (4.3) | 5^a^ | 10^a^ |  |
|  | hypertrophy | 56 | (24) | *9.3* | 6.0 | *5.3* | (2.6) | **4.9** | (2.6) |  |  |  |
|  |  |  |  |  |  |  |  |  |  |  |  |  |
| **BB (mkd)** | absolute liver weight | 426 | (368) | *NC* | | **47** | (26) | *85* | (65) | 200^b^ | 300^b^ |  |
|  | hypertrophy | *228* | (197) | *202* | (160) | 240 | (194) | 199 | (177) |  |  |  |
|  |  |  |  |  |  |  |  |  |  |  |  |  |
| **TCP (mkd)** | absolute liver weight | *92* | (54) | *17* | (11) | 36 | (28) | 7.4 | (4.8) | 10^c^ | 25^c^ |  |
|  | vacuolation | NA | | 23 | (15) | *6.8* | (1.5) | 8.0 | (6.5) |  |  |  |
|  | hypertrophy | 100 | (82) | 45 | (29) | 23 | (14) | **4.5** | (1.0) |  |  |  |
|  | necrosis, single cell | 131 | (64) | 39 | (19) | 42 | (34) | 38 | (21) |  |  |  |
|  |  |  |  |  |  |  |  |  |  |  |  |  |
| **MDA (ppm)** | absolute thyroid weight | 294 | (218) | 328 | (220) | 218 | (156) | NC | | 200^d^ | 375^d^ |  |
|  | follicular cell hypertrophy | *221* | (137) | *148* | *(47)* | **43** | (12) | *169* | (63) |  |  |  |
|  | follicular cell hyperplasia | 719 | (535) | 228 | (192) | 49 | (35) | 189 | (98) |  |  |  |
|  |  |  |  |  |  |  |  |  |  |  |  |  |
| **NDPA (ppm)** | absolute bladder weight | *1841* | (1460) | NC | | 2058 | (1673) | NC | | 1000^e^ | 2000^e^ |  |
|  | increased mitosis | *1980* | (1568) | 2879 | (1638) | 2581 | (1900) | NA | |  |  |  |
|  | diffuse transitional epithelial hyperplasia | NA | | 3625 | (2680) | *1689* | (1053) | **1567** | (971) |  |  |  |
|  | increased necrosis epithelial cell | NA | | *2559* | (1529) | 2876 | (2414) | 3838 | (3017) |  |  |  |
|  |  |  |  |  |  |  |  |  |  |  |  |  |
| **HZB (ppm)** | absolute liver weight | NC* | | NC* | | NC* | | NC* | | 80^f^ | 200^f^ |  |
|  | hypertrophy | NA | | NA | | NA | | 151 | (76) |  |  |  |
|  | Microvesiculation | NA | | NA | | NA | | 199 | (176) |  |  |  |
|  | bile duct duplication | NA | | NA | | NA | | **55** | (37) |  |  |  |

^a^(Dodd et al. 2012d)^, b^ (Dodd et al. 2013b), ^c^ (Dodd et al. 2012a), ^d^ (Dodd et al. 2012c), ^e^ (Dodd et al. 2013a), ^f^ (Dodd et al. 2012b)

The POD values for TBB, BB and TCP are in mg/kg and for MDA, NDPA, and HZB in ppm. The lowest apical responses for each time point and across all time points are shown in italic and bold fonts, respectively.

Not Available (NA): no finding; Not Calculated (NC): no BMD_a_ can be calculated; failed BMD_a_ modeling; NC*: BMD_a_>the highest dose

**Table 4.** The number of significantly differentially expressed genes (FC ≥ 1.5; FDR *p ≤* 0.05) in rats following exposure to TBB, BB, TCP, MDA, NDPA, and HZB for the 5, 14, 28, and 90 day time points .

****cd: concentration-dependent; td: time-dependent.

**Table 5.** The average of the BMD_t_/POD ratios, Pearson's correlation coefficient *r* with *p*-value for transcritional BMD(L) _t_ vs. apical PODs, and likelihood ratio chi-square and *p*-value for the 11 transcriptional approaches at the 5 day time point.

Check marks (√) and exclamation marks (!) indicate whether an approach met or failed to meet the criteria, respectively.

**Table 6.** Assessment of each approach against the three criteria for predicting apical PODs at the 5, 14, 28, and 90 day time points.

| **panel A- BMD_t_ vs. NOAEL** | | | | |  | **panel C - BMD_t_ vs. lowest apical BMD_a_**  **(time point matched)** | | | | |
| --- | --- | --- | --- | --- | --- | --- | --- | --- | --- | --- |
| approach | 5 day | 14 day | 28 day | 90 day |  | approach | 5 day | 14 day | 28 day | 90 day |
| **1** | **!** | **√** | **√** | **!** |  | **1** | **√** | **√** | **!** | **!** |
| **2** | **!** | **!** | **!** | **!** |  | **2** | **√** | **√** | **!** | **!** |
| **3** | **!** | **!** | **!** | **!** |  | **3** | **√** | **√** | **!** | **!** |
| **4** | **√** | **√** | **!** | **√** |  | **4** | **√** | **√** | **√** | **√** |
| **5** | **!** | **√** | **!** | **!** |  | **5** | **√** | **√** | **!** | **!** |
| **6** | **!** | **√** | **!** | **!** |  | **6** | **√** | **√** | **!** | **!** |
| **7** | **!** | **√** | **√** | **!** |  | **7** | **√** | **√** | **!** | **!** |
| **8** | **!** | **!** | **!** | **!** |  | **8** | **√** | **√** | **!** | **!** |
| **9** | **!** | **!** | **√** | **!** |  | **9** | **!** | **!** | **!** | **!** |
| **10** | **!** | **!** | **!** | **!** |  | **10** | **!** | **!** | **!** | **!** |
| **11** | **!** | **!** | **!** | **!** |  | **11** | **!** | **!** | **!** | **!** |
| **panel B - BMD_t_ vs. LOAEL** | | | | |  | **panel D - BMD_t_ vs. lowest apical BMD_a_**  **across any time point** | | | | |
| approach | 5 day | 14 day | 28 day | 90 day |  | approach | 5 day | 14 day | 28 day | 90 day |
| **1** | **√** | **√** | **√** | **!** |  | **1** | **!** | **!** | **!** | **!** |
| **2** | **!** | **√** | **√** | **!** |  | **2** | **!** | **!** | **!** | **!** |
| **3** | **!** | **!** | **√** | **!** |  | **3** | **!** | **!** | **!** | **!** |
| **4** | **√** | **√** | **!** | **√** |  | **4** | **!** | **!** | **√** | **!** |
| **5** | **√** | **√** | **√** | **√** |  | **5** | **!** | **!** | **!** | **!** |
| **6** | **√** | **√** | **√** | **!** |  | **6** | **!** | **!** | **!** | **!** |
| **7** | **√** | **√** | **√** | **!** |  | **7** | **!** | **!** | **!** | **!** |
| **8** | **√** | **√** | **√** | **√** |  | **8** | **!** | **!** | **!** | **!** |
| **9** | **!** | **!** | **√** | **!** |  | **9** | **!** | **!** | **!** | **!** |
| **10** | **!** | **!** | **√** | **!** |  | **10** | **!** | **!** | **!** | **!** |
| **11** | **!** | **!** | **√** | **!** |  | **11** | **!** | **!** | **!** | **!** |
| **panel E - BMDL_t_ vs. NOAEL** | | | | |  | **panel F - BMDL_t_ vs. LOAEL** | | |  |  |
| approach | 5 day | 14 day | 28 day | 90 day |  | approach | 5 day | 14 day | 28 day | 90 day |
| **1** | **√** | **√** | **!** | **!** |  | **1** | **√** | **√** | **√** | **!** |
| **2** | **!** | **√** | **√** | **!** |  | **2** | **√** | **√** | **√** | **!** |
| **3** | **!** | **!** | **√** | **!** |  | **3** | **!** | **!** | **√** | **!** |
| **4** | **√** | **!** | **!** | **√** |  | **4** | **√** | **!** | **!** | **!** |
| **5** | **√** | **√** | **!** | **√** |  | **5** | **√** | **√** | **√** | **√** |
| **6** | **√** | **√** | **√** | **!** |  | **6** | **√** | **√** | **√** | **!** |
| **7** | **√** | **√** | **√** | **!** |  | **7** | **√** | **√** | **√** | **!** |
| **8** | **√** | **√** | **!** | **√** |  | **8** | **√** | **√** | **!** | **√** |
| **9** | **!** | **!** | **√** | **!** |  | **9** | **!** | **!** | **√** | **!** |
| **10** | **!** | **!** | **√** | **!** |  | **10** | **!** | **!** | **√** | **!** |
| **11** | **!** | **!** | **√** | **!** |  | **11** | **!** | **!** | **√** | **!** |

Check marks (√) and exclamation points (!) indicate whether an approach met or did not meet the three criteria, respectively.

**Table 7.** Assessment of BMD_t_ and BMDL_t_ derived from each approach against the three criteria for predicting apical PODs at the 5, 14, 28, and 90 day time points.

| **Approach** | **5 days** | **14 days** | **28 days** | **90 days** | **sum** | **percent** |
| --- | --- | --- | --- | --- | --- | --- |
| **1** | 15 | 16 | 14 | 10 | 55 | 76% |
| **2** | 13 | 15 | 14 | 9 | 51 | 71% |
| **3** | 11 | 11 | 13 | 10 | 45 | 63% |
| **4** | 17 | 15 | 13 | 15 | 60 | 83% |
| **5** | 14 | 16 | 13 | 12 | 55 | 76% |
| **6** | 13 | 16 | 14 | 8 | 51 | 71% |
| **7** | 15 | 16 | 15 | 9 | 55 | 76% |
| **8** | 15 | 15 | 12 | 11 | 53 | 74% |
| **9** | 11 | 10 | 15 | 8 | 44 | 61% |
| **10** | 9 | 9 | 12 | 8 | 38 | 53% |
| **11** | 9 | 9 | 12 | 8 | 38 | 53% |

BMD_t_ values were assessed for predicting four apical endpoints (NOAEL, LOAEL, lowest BMD_a_ at matched time point of apical endpoints, lowest BMD_a_ overall of apical data); BMDL_t_ values were assessed for predicting two apical endpoint (NOAEL and LOAEL). Sum = (3 criteria x 4 apical PODs x 4 time points=48) + (3 criteria x 2 apical PODs x 4 time points=24) = 72. The top approaches that met the three criteria are highlighted in blue (there was a 3-way tie for second place).

**Figure 1.**


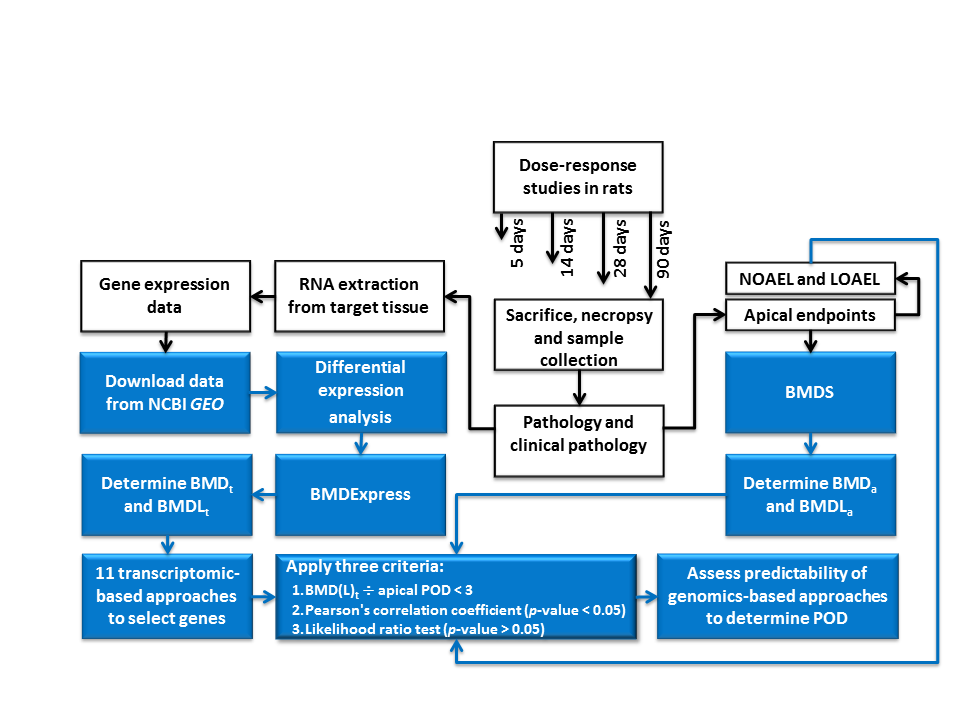


**Figure 2.**

**Figure 3.
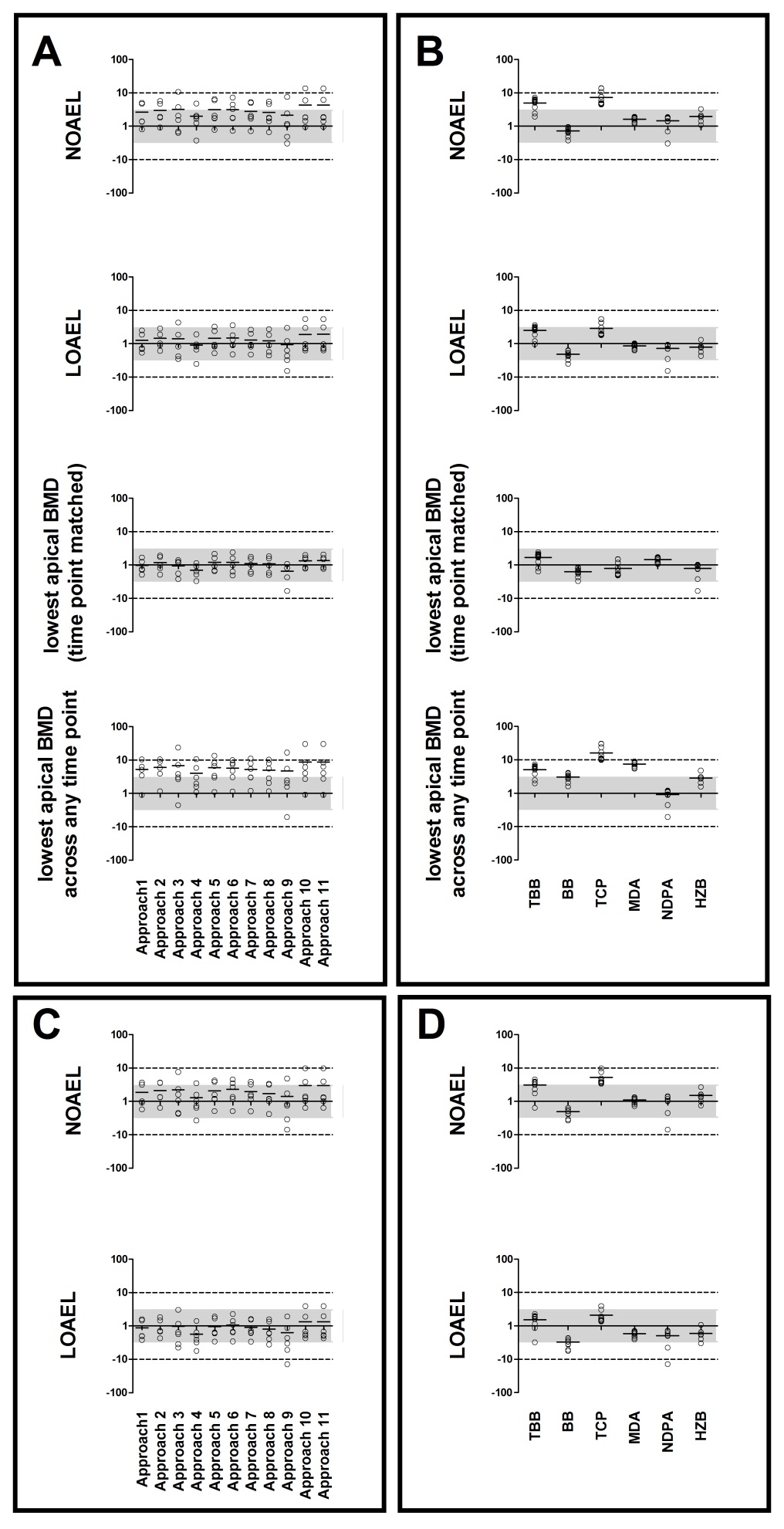
**

**SUPPLEMENTARY MATERIAL**

**Figure S1.**

**
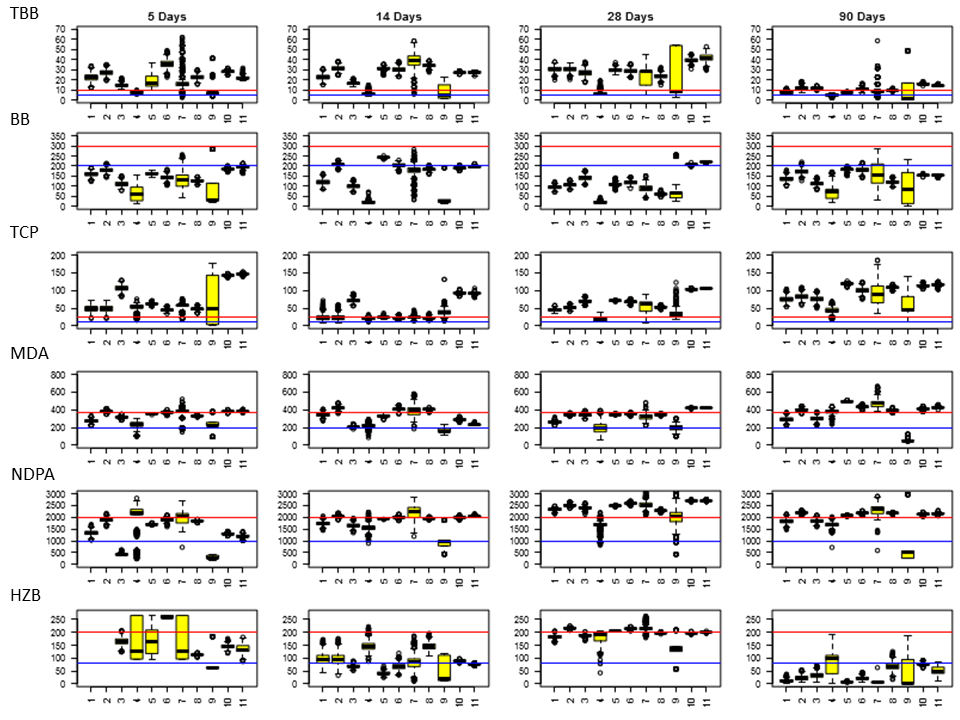
**

**Figure S2.**

**Figure S3.**

**Figure S4.**

**
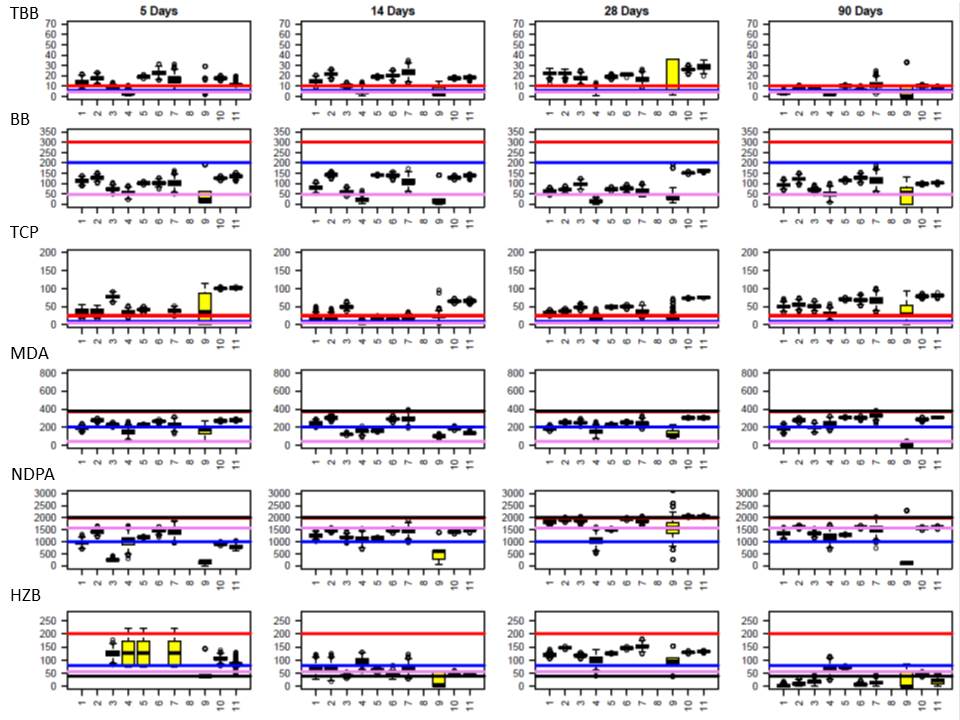
**

**Figure S5.**


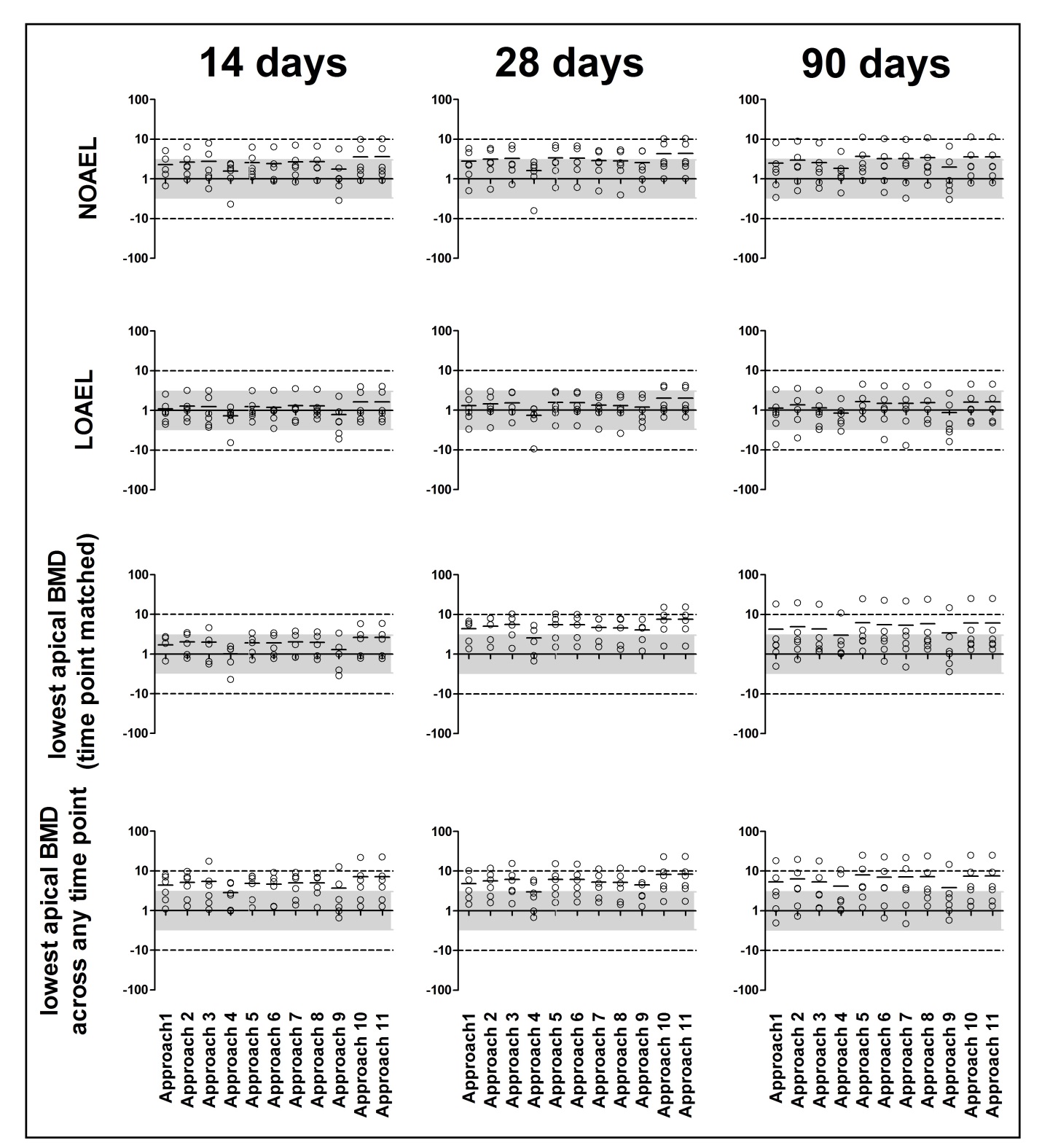


**Figure S6**


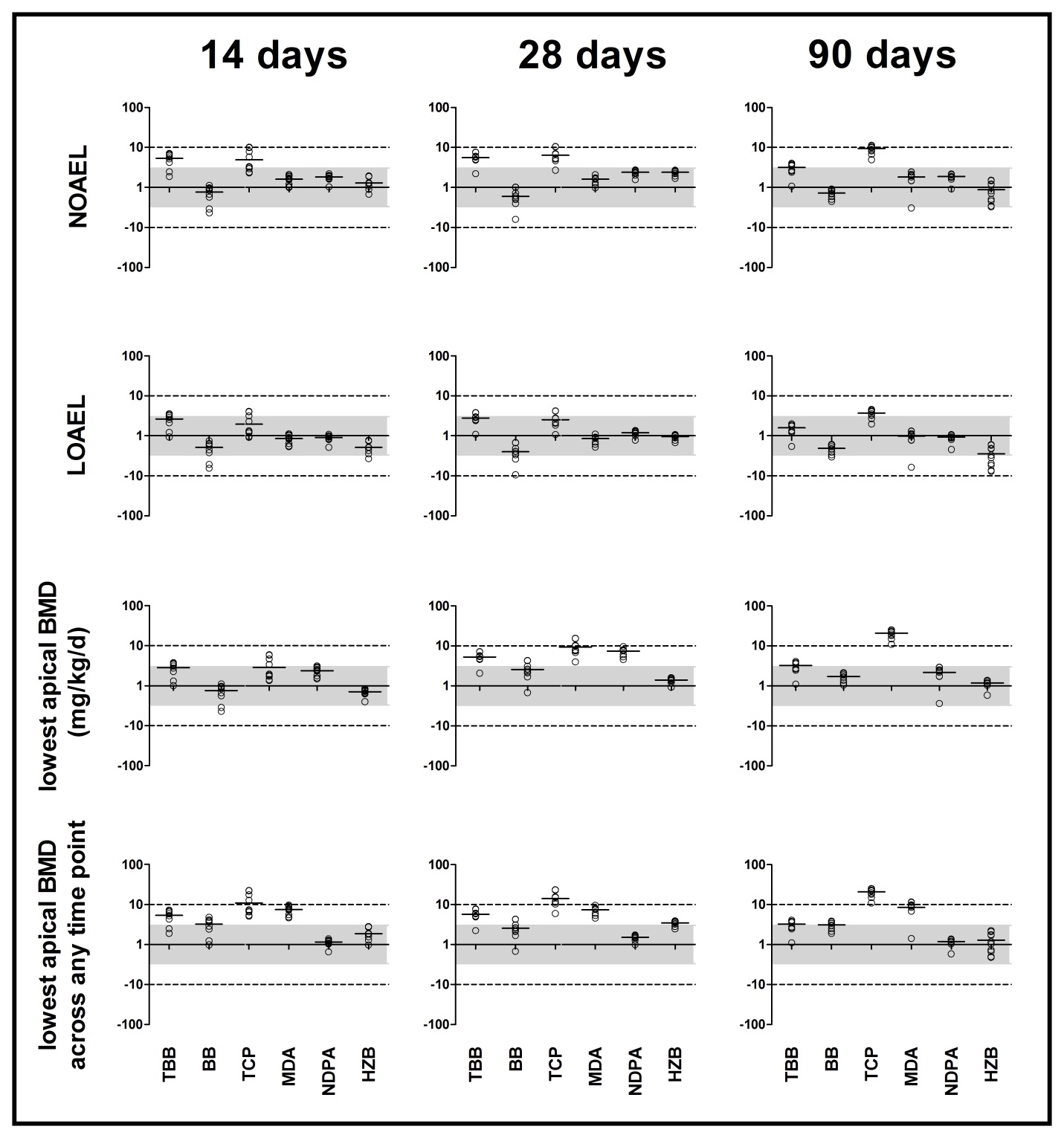


**Figure S7.**


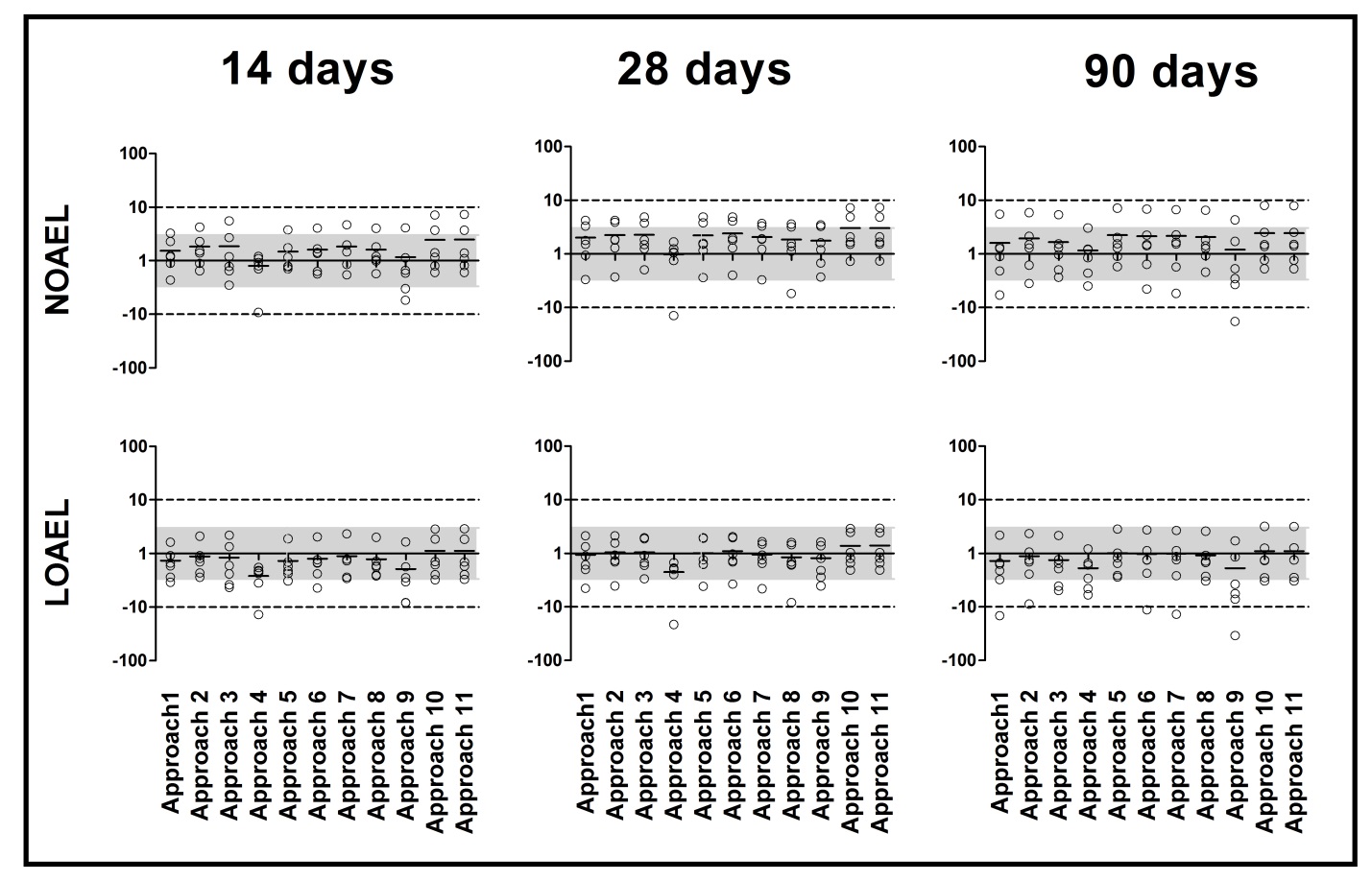


**Figure S8.**

**
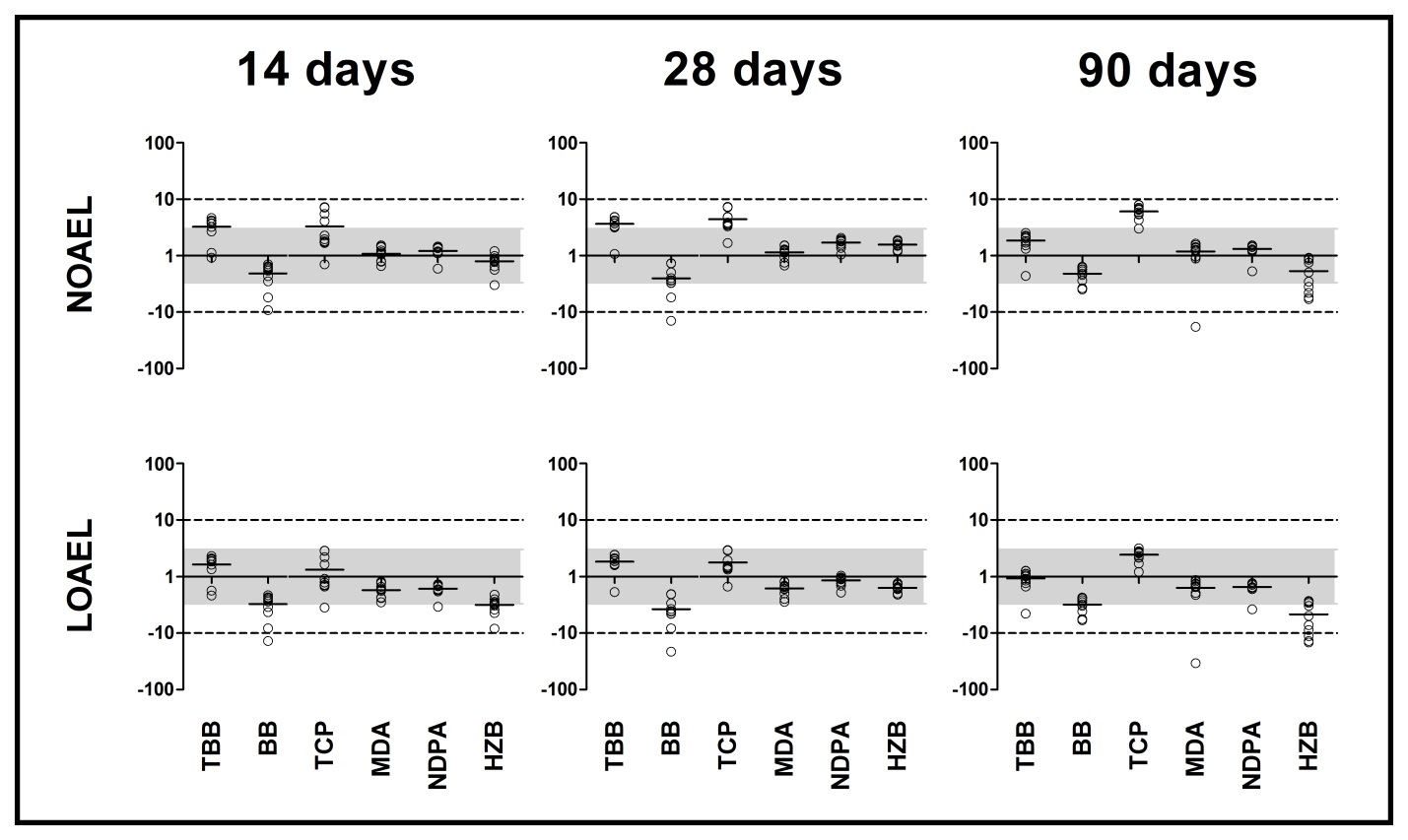
**

**Figure S9.**

**
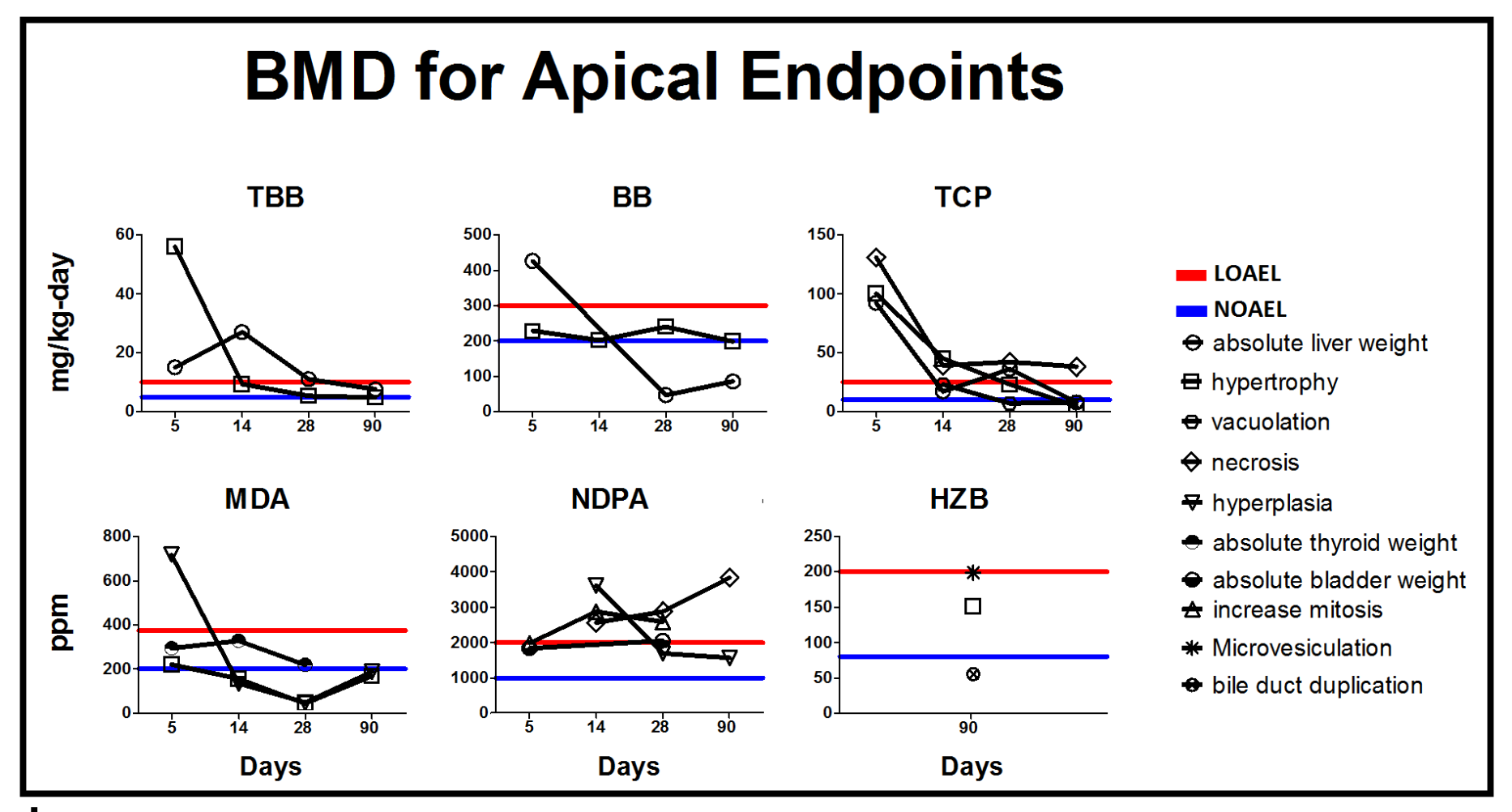
**

**Figure S10.**


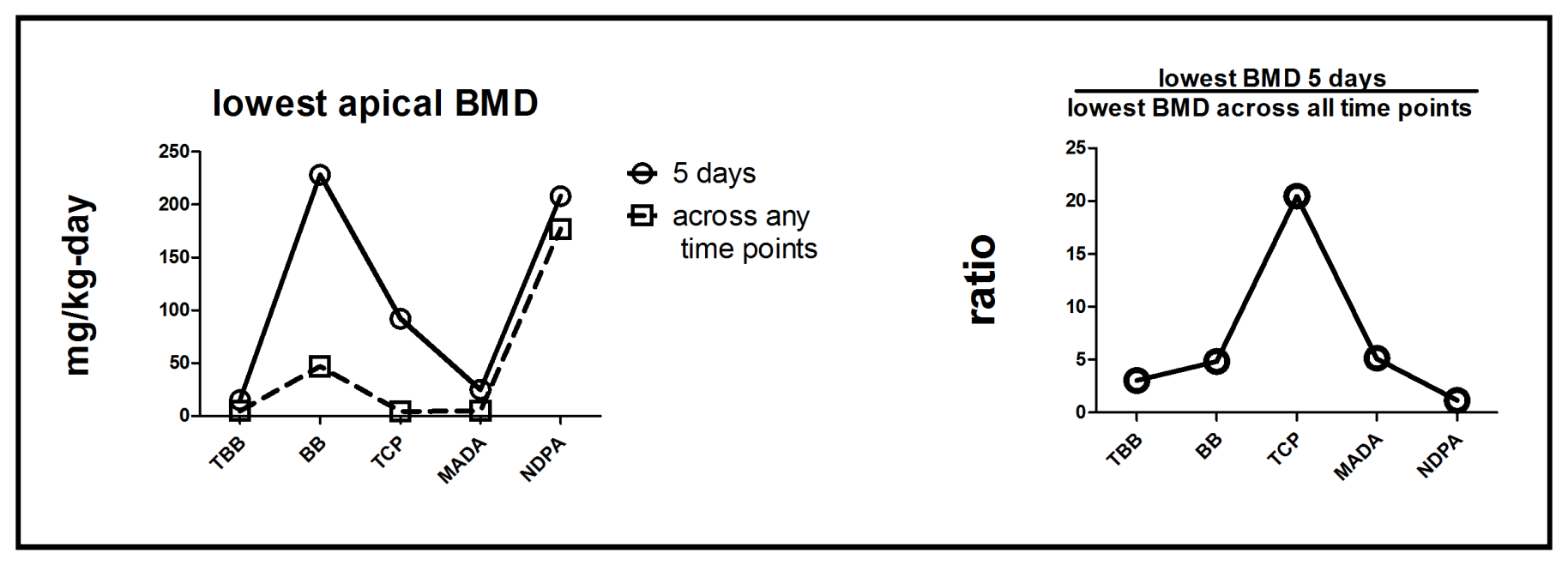


**Figure S11.**

**
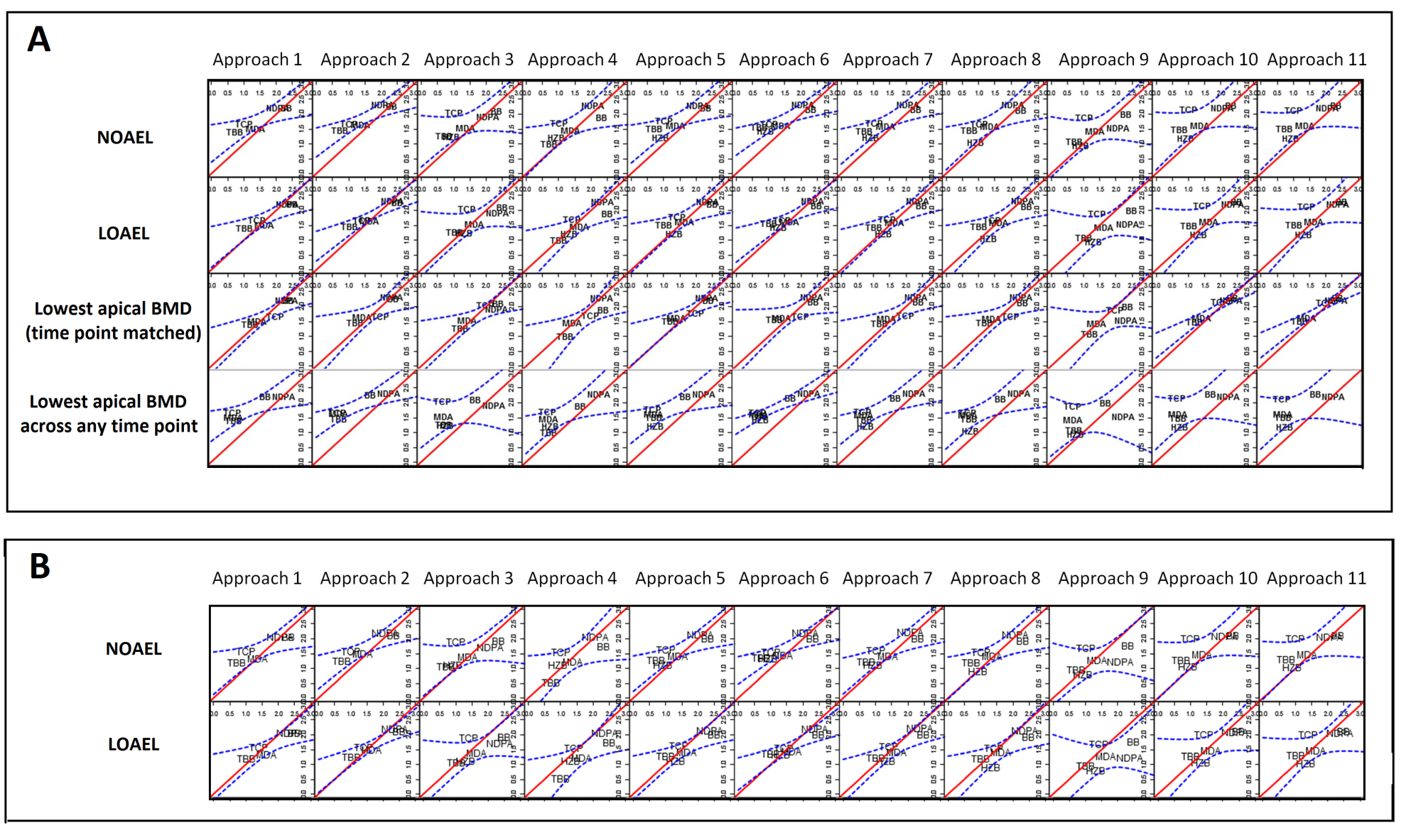
**


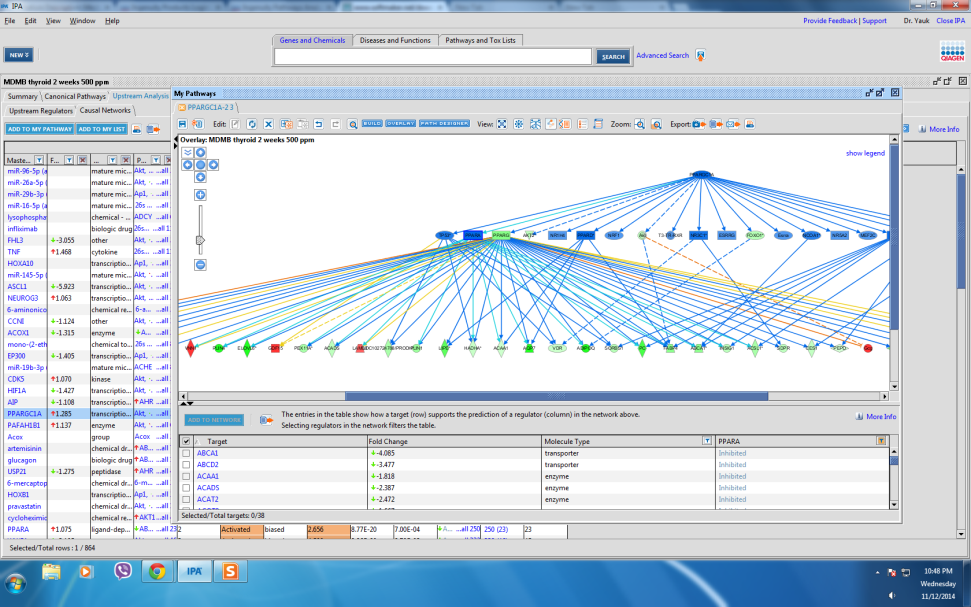
**Figure S12.
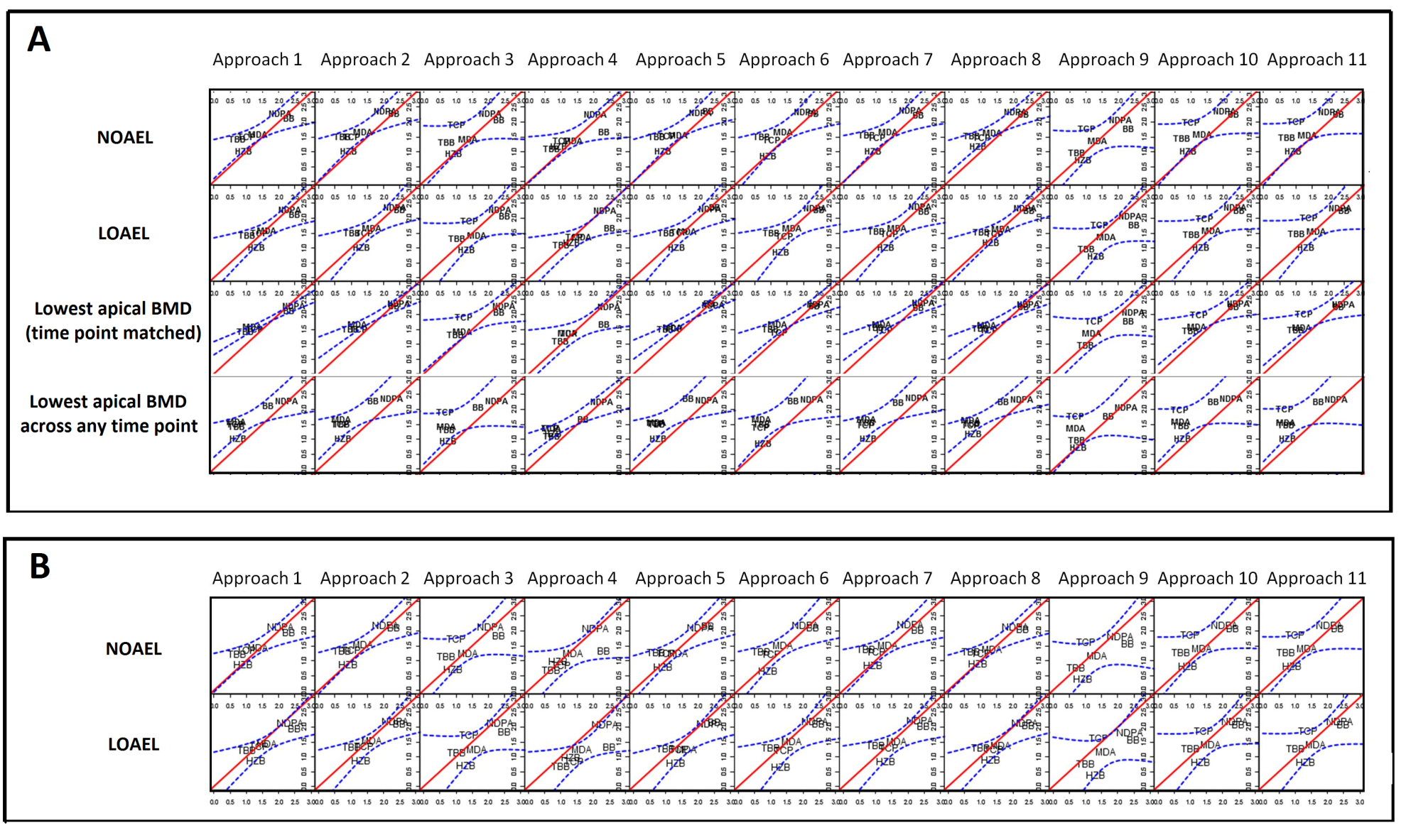
**

**Figure 1.** Overview of study method.

**Figure S13.**

**
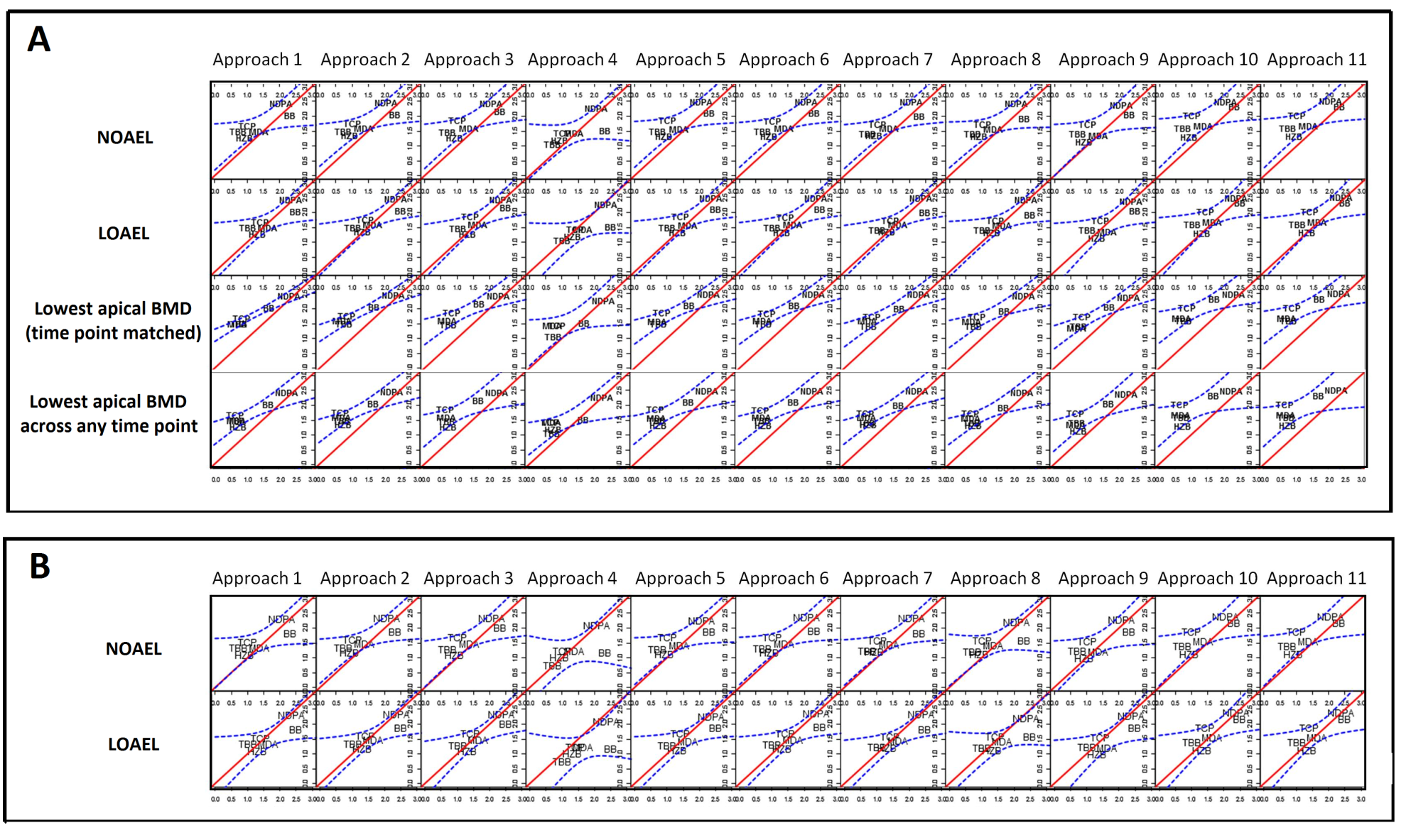
**

**Figure S14.**

**
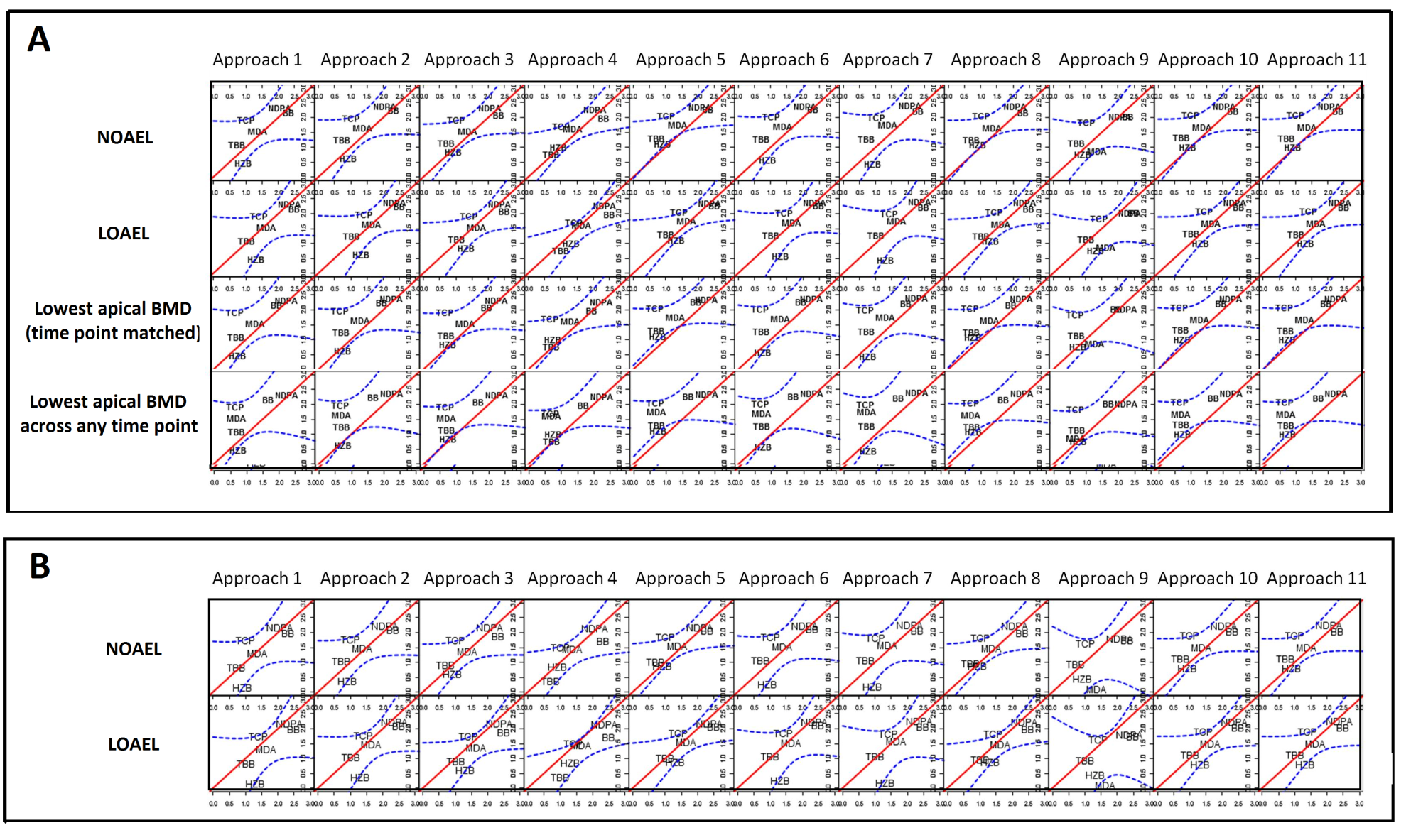
**

**Figure S15.**

**
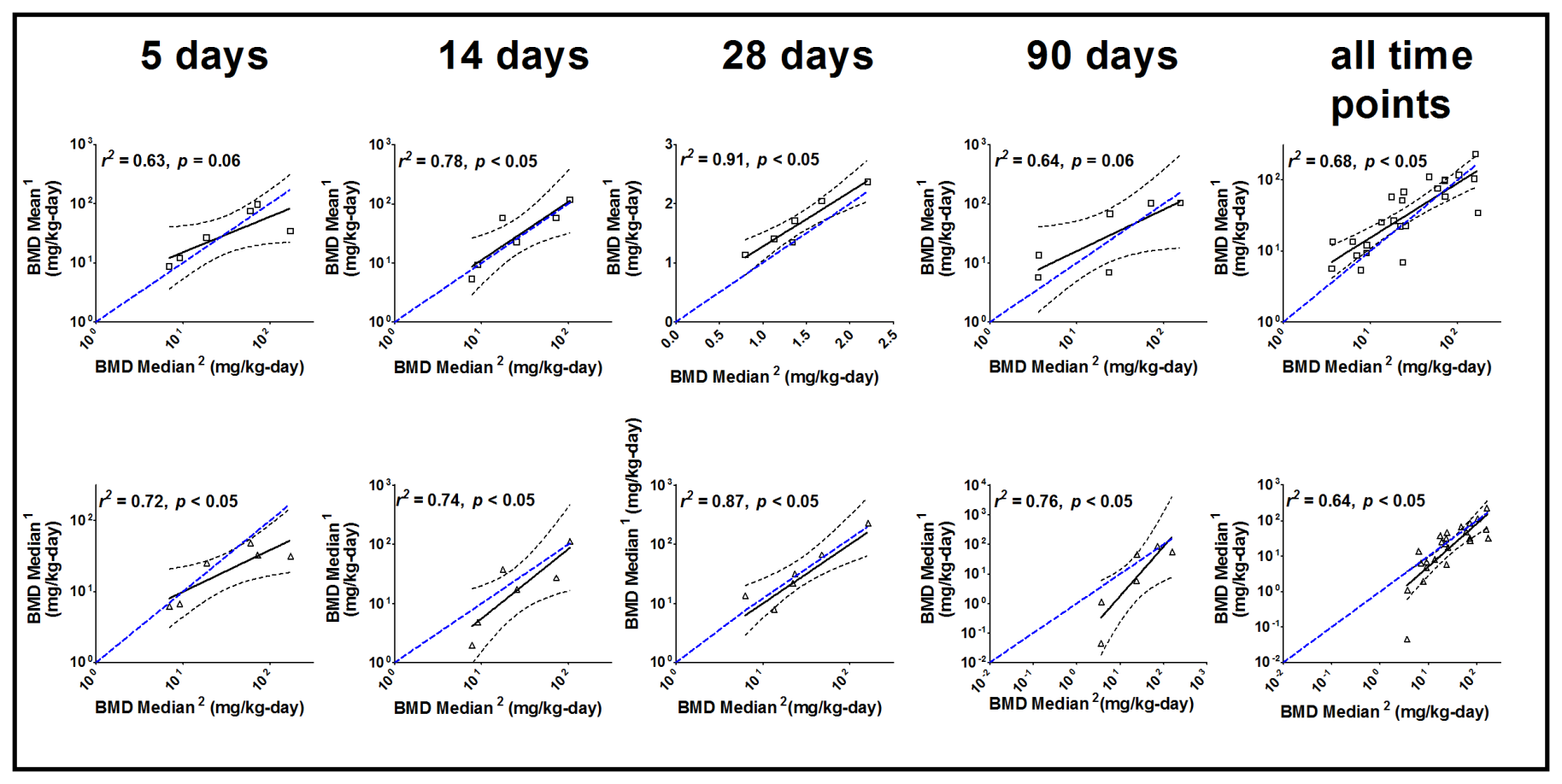
**

**Figure S16.
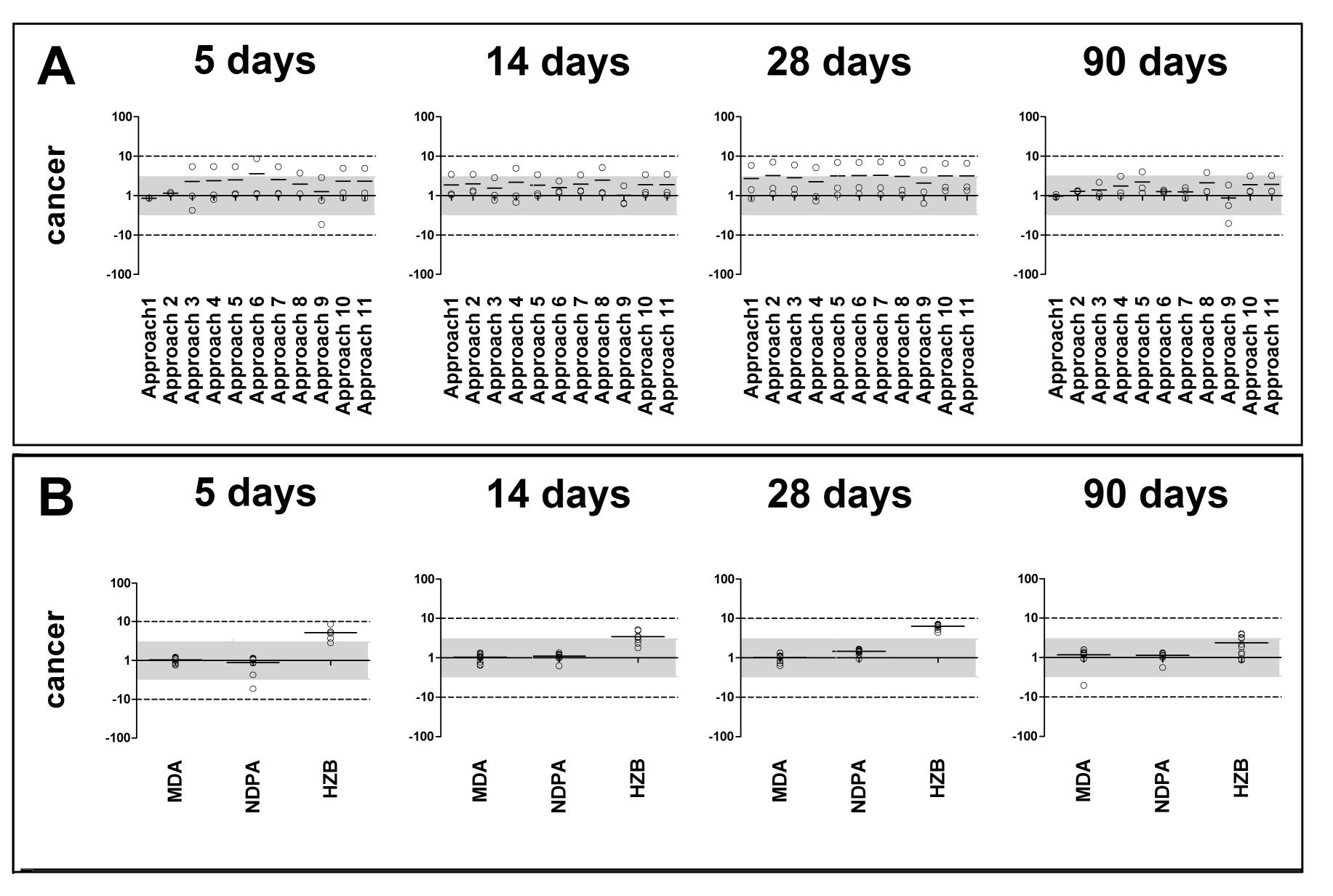
**

References

Andersen ME, Clewell HJ,3rd, Bermudez E, Dodd DE, Willson GA, Campbell JL, Thomas RS (2010) Formaldehyde: integrating dosimetry, cytotoxicity, and genomics to understand dose-dependent transitions for an endogenous compound . Toxicol Sci 118:716-731. doi: 10.1093/toxsci/kfq303 [doi]

Auerbach SS, Phadke DP, Mav D, Holmgren S, Gao Y, Xie B, Shin JH, Shah RR, Merrick BA, Tice RR (2015) RNA-Seq-based toxicogenomic assessment of fresh frozen and formalin-fixed tissues yields similar mechanistic insights . J Appl Toxicol 35:766-780. doi: 10.1002/jat.3068 [doi]

Barton-Maclaren T, Westphal M, Sarwar E, Mattison D, Chiu W, Dix D, Kavlock R, Krewski D (2016) Challenges and opportunities in the risk assessment of existing substances in Canada: lessons learned from the international community

Benjamini Y, Hochberg Y (2007) Controlling the False Discovery Rate: A Practical and Powerful Approach to Multiple Testing . Journal of the Royal Statistical Society.Series B (Methodological) 57:10-300. doi: 10.2307/2346101

Bercu JP, Jolly RA, Flagella KM, Baker TK, Romero P, Stevens JL (2010) Toxicogenomics and cancer risk assessment: a framework for key event analysis and dose-response assessment for nongenotoxic carcinogens . Regul Toxicol Pharmacol 58:369-381. doi: 10.1016/j.yrtph.2010.08.002 [doi]

Bhat VS, Hester SD, Nesnow S, Eastmond DA (2013) Concordance of transcriptional and apical benchmark dose levels for conazole-induced liver effects in mice . Toxicol Sci 136:205-215. doi: 10.1093/toxsci/kft182 [doi]

Black MB, Parks BB, Pluta L, Chu TM, Allen BC, Wolfinger RD, Thomas RS (2014) Comparison of microarrays and RNA-seq for gene expression analyses of dose-response experiments . Toxicol Sci 137:385-403. doi: 10.1093/toxsci/kft249 [doi]

Black MB, Budinsky RA, Dombkowski A, Cukovic D, LeCluyse EL, Ferguson SS, Thomas RS, Rowlands JC (2012) Cross-species comparisons of transcriptomic alterations in human and rat primary hepatocytes exposed to 2,3,7,8-tetrachlorodibenzo-p-dioxin . Toxicol Sci 127:199-215. doi: 10.1093/toxsci/kfs069 [doi]

Bourdon JA, Williams A, Kuo B, Moffat I, White PA, Halappanavar S, Vogel U, Wallin H, Yauk CL (2013) Gene expression profiling to identify potentially relevant disease outcomes and support human health risk assessment for carbon black nanoparticle exposure . Toxicology 303:83-93. doi: 10.1016/j.tox.2012.10.014 [doi]

Bourdon-Lacombe JA, Moffat ID, Deveau M, Husain M, Auerbach S, Krewski D, Thomas RS, Bushel PR, Williams A, Yauk CL (2015) Technical guide for applications of gene expression profiling in human health risk assessment of environmental chemicals . Regul Toxicol Pharmacol 72:292-309. doi: 10.1016/j.yrtph.2015.04.010 [doi]

Chepelev NL, Long AS, Williams A, Kuo B, Gagné R, Kennedy DA, Phillips DH, Arlt VM, White PA, Yauk CL (2016; 2015) Transcriptional Profiling of Dibenzo[def,p]chrysene-induced Spleen Atrophy Provides Mechanistic Insights into its Immunotoxicity in MutaMouse . Toxicological Sciences 149:251 <last_page> 268. doi: 10.1093/toxsci/kfv232

Chepelev NL, Long AS, Bowers WJ, Gagné R, Williams A, Kuo B, Phillips DH, Arlt VM, White PA, Yauk CL (2016; 2016) Transcriptional profiling of the mouse hippocampus supports an NMDAR-mediated neurotoxic mode of action for benzo[a]pyrene . Environ Mol Mutagen. doi: 10.1002/em.22020

Chepelev NL, Moffat ID, Labib S, Bourdon-Lacombe J, Kuo B, Buick JK, Lemieux F, Malik AI, Halappanavar S, Williams A, Yauk CL (2015) Integrating toxicogenomics into human health risk assessment: Lessons learned from the benzo[a]pyrene case study . Crit Rev Toxicol 45:44 <last_page> 52. doi: 10.3109/10408444.2014.973935

Clewell HJ, Thomas RS, Kenyon EM, Hughes MF, Adair BM, Gentry PR, Yager JW (2011) Concentration- and time-dependent genomic changes in the mouse urinary bladder following exposure to arsenate in drinking water for up to 12 weeks . Toxicol Sci 123:421-432. doi: 10.1093/toxsci/kfr199 [doi]

Committee on Toxicity Testing and Assessment of Environmental Agents (2007) Toxicity Testing in the 21st Century: A Vision and a Strategy . National Academies Press

Cote I, Andersen ME, Ankley GT, Barone S, Birnbaum LS, Boekelheide K, Bois FY, Burgoon LD, Chiu WA, Crawford-Brown D, Crofton KM, DeVito M, Devlin RB, Edwards SW, Guyton KZ, Hattis D, Judson RS, Knight D, Krewski D, Lambert J, Maull EA, Mendrick D, Paoli GM, Patel CJ, Perkins EJ, Poje G, Portier CJ, Rusyn I, Schulte PA, Simeonov A, Smith MT, Thayer KA, Thomas RS, Thomas R, Tice RR, Vandenberg JJ, Villeneuve DL, Wesselkamper S, Whelan M, Whittaker C, White R, Xia M, Yauk C, Zeise L, Zhao J, DeWoskin RS (2016) The Next Generation of Risk Assessment Multiyear Study- Highlights of Findings, Applications to Risk Assessment and Future Directions . Environ Health Perspect. doi: 10.1289/EHP233 [doi]

Council of Canadian Academies Integrating Emerging Technologies into Chemical Safety Assessment. <http://www.scienceadvice.ca/en/assessments/completed/pesticides.aspx>. Accessed 1/18/2016 2016

Cui X, Hwang JT, Qiu J, Blades NJ, Churchill GA (2005) Improved statistical tests for differential gene expression by shrinking variance components estimates . Biostatistics 6:59-75. doi: 6/1/59 [pii]

Davis JA, Gift JS, Zhao QJ (2011) Introduction to benchmark dose methods and U.S. EPA's benchmark dose software (BMDS) version 2.1.1 . Toxicol Appl Pharmacol 254:181-191. doi: 10.1016/j.taap.2010.10.016 [doi]

Dodd DE, Pluta LJ, Sochaski MA, Banas DA, Thomas RS (2012a) Subchronic hepatotoxicity evaluation of 2,3,4,6-tetrachlorophenol in sprague dawley rats . J Toxicol 2012:376246. doi: 10.1155/2012/376246 [doi]

Dodd DE, Pluta LJ, Sochaski MA, Wall HG, Thomas RS (2012b) Subchronic hepatotoxicity evaluation of hydrazobenzene in Fischer 344 rats . Int J Toxicol 31:564-571. doi: 10.1177/1091581812465322 [doi]

Dodd DE, Pluta LJ, Sochaski MA, Funk KA, Thomas RS (2012c) Subchronic thyroid toxicity evaluation of 4,4'-methylenebis(N,N'-dimethyl)aniline in Fischer 344 rats . J Toxicol Environ Health A 75:637-648. doi: 10.1080/15287394.2012.688481 [doi]

Dodd DE, Pluta LJ, Sochaski MA, Funk KA, Thomas RS (2012d) Subchronic hepatotoxicity evaluation of 1,2,4-tribromobenzene in Sprague-Dawley rats . Int J Toxicol 31:250-256. doi: 10.1177/1091581812437974 [doi]

Dodd DE, Pluta LJ, Sochaski MA, Funk KA, Thomas RS (2013a) Subchronic urinary bladder toxicity evaluation of N-Nitrosodiphenylamine in Fischer 344 rats . J Appl Toxicol 33:383-389. doi: 10.1002/jat.2798 [doi]

Dodd DE, Pluta LJ, Sochaski MA, Banas DA, Thomas RS (2013b) Subchronic hepatotoxicity evaluation of bromobenzene in Fischer 344 rats . Journal of Applied Toxicology 33:370 <last_page> 377. doi: 10.1002/jat.2732

Dong H, Gill S, Curran IH, Williams A, Kuo B, Wade MG, Yauk CL (2015) Toxicogenomic assessment of liver responses following subchronic exposure to furan in Fischer F344 rats . Arch Toxicol. doi: 10.1007/s00204-015-1561-2 [doi]

Efron B TR (1993) An introduction to the bootstrap. . Chapman & Hall, New York

Firestone M, Kavlock R, Zenick H, Kramer M, US Environmental Protection Agency Working Group on the Future of Toxicity Testing (2010) The U.S. Environmental Protection Agency strategic plan for evaluating the toxicity of chemicals . J Toxicol Environ Health B Crit Rev 13:139-162. doi: 10.1080/10937404.2010.483178 [doi]

Guyton KZ, Kyle AD, Aubrecht J, Cogliano VJ, Eastmond DA, Jackson M, Keshava N, Sandy MS, Sonawane B, Zhang L, Waters MD, Smith MT (2009) Improving prediction of chemical carcinogenicity by considering multiple mechanisms and applying toxicogenomic approaches . Mutat Res 681:230-240. doi: 10.1016/j.mrrev.2008.10.001 [doi]

Hester S, Eastmond DA, Bhat VS (2015) Developing toxicogenomics as a research tool by applying benchmark dose-response modelling to inform chemical mode of action and tumorigenic potency . Int J Biotechnol 14:28. doi: 10.1504/IJBT.2015.074796

<http://www.epa.gov/iris>

Irizarry RA, Hobbs B, Collin F, Beazer-Barclay YD, Antonellis KJ, Scherf U, Speed TP (2003) Exploration, normalization, and summaries of high density oligonucleotide array probe level data . Biostatistics 4:249-264. doi: 10.1093/biostatistics/4.2.249 [doi]

Jackson AF, Williams A, Recio L, Waters MD, Lambert IB, Yauk CL (2014) Case study on the utility of hepatic global gene expression profiling in the risk assessment of the carcinogen furan . Toxicol Appl Pharmacol 274:63-77. doi: 10.1016/j.taap.2013.10.019 [doi]

Krewski D, Westphal M, Andersen ME, Paoli GM, Chiu WA, Al-Zoughool M, Croteau MC, Burgoon LD, Cote I (2014) A framework for the next generation of risk science . Environ Health Perspect 122:796-805. doi: 10.1289/ehp.1307260 [doi]

Kuo B, Francina Webster A, Thomas RS, Yauk CL (2015) BDExpress Data Viewer - a visualization tool to analyze BMDExpress datasets  . J Appl Toxicol DOI 10.1002/jat.3265

Labib S, Williams A, Guo CH, Leingartner K, Arlt VM, Schmeiser HH, Yauk CL, White PA, Halappanavar S (2015) Comparative transcriptomic analyses to scrutinize the assumption that genotoxic PAHs exert effects via a common mode of action . Arch Toxicol. doi: 10.1007/s00204-015-1595-5 [doi]

Moffat I, Chepelev NL, Labib S, Bourdon-Lacombe J, Kuo B, Buick JK, Lemieux F, Williams A, Halappanavar S, Malik AI, Luijten M, Aubrecht J, Hyduke DR, Fornace AJ,Jr, Swartz CD, Recio L, Yauk CL (2015) Comparison of toxicogenomics and traditional approaches to inform mode of action and points of departure in human health risk assessment of benzo[a]pyrene in drinking water . Crit Rev Toxicol 45:1-43. doi: 10.3109/10408444.2014.973934 [doi]

NRC (2007) Applications of toxicogenomic technologies to predictive toxicology and risk assessment . National Academies Press, Washington, D.C.

NRC (2009) Science and Decisions: Advancing Risk Assessment . National Academies Press

NRC (2010) Toxicity Pathway-Based Risk Assessment: Preparing for Paradigm Change: A Symposium Summary . National Academies Press

NTP (1978) Bioassay of Hydrazobenzene for Possible Carcinogenicity. 92 U S Department of Health and Human Services National Toxicology Program, Washington, DC

NTP (1979a) Bioassay of 4,4’-Methylenebis-(N,N-Dimethyl)Benzenamine for Possible Carcinogenicity. 186 U S Department of Health and Human Services National Toxicology Program, Washington, DC

NTP (1979b) Bioassay of N-Nitrosodiphenylamine for Possible Carcinogenicity. 164 U S Department of Health and Human Services National Toxicology Program, Washington, DC

Searle SR, Speed FM, Milliken GA (2012; 1980) Population Marginal Means in the Linear Model: An Alternative to Least Squares Means . The American Statistician 34:216 <last_page> 221. doi: 10.1080/00031305.1980.10483031

Thomas RS, Clewell HJ,3rd, Allen BC, Yang L, Healy E, Andersen ME (2012) Integrating pathway-based transcriptomic data into quantitative chemical risk assessment: a five chemical case study . Mutat Res 746:135-143. doi: 10.1016/j.mrgentox.2012.01.007 [doi]

Thomas RS, Allen BC, Nong A, Yang L, Bermudez E, Clewell HJ, Andersen ME (2007) A Method to Integrate Benchmark Dose Estimates with Genomic Data to Assess the Functional Effects of Chemical Exposure . Toxicological Sciences 98:240 <last_page> 248. doi: 10.1093/toxsci/kfm092

Thomas RS, Clewell HJ,3rd, Allen BC, Wesselkamper SC, Wang NC, Lambert JC, Hess-Wilson JK, Zhao QJ, Andersen ME (2011) Application of transcriptional benchmark dose values in quantitative cancer and noncancer risk assessment . Toxicol Sci 120:194-205. doi: 10.1093/toxsci/kfq355 [doi]

Thomas RS, Rank DR, Penn SG, Zastrow GM, Hayes KR, Pande K, Glover E, Silander T, Craven MW, Reddy JK, Jovanovich SB, Bradfield CA (2001) Identification of toxicologically predictive gene sets using cDNA microarrays . Mol Pharmacol 60:1189-1194

Thomas RS, Wesselkamper SC, Wang NCY, Zhao QJ, Petersen DD, Lambert JC, Cote I, Yang L, Healy E, Black MB, Clewell HJ, Allen BC, Andersen ME (2013a) Temporal Concordance Between Apical and Transcriptional Points of Departure for Chemical Risk Assessment . Toxicol Sci 134:180 <last_page> 194. doi: 10.1093/toxsci/kft094

Thomas RS, Philbert MA, Auerbach SS, Wetmore BA, Devito MJ, Cote I, Rowlands JC, Whelan MP, Hays SM, Andersen ME, Meek ME, Reiter LW, Lambert JC, Clewell HJ,3rd, Stephens ML, Zhao QJ, Wesselkamper SC, Flowers L, Carney EW, Pastoor TP, Petersen DD, Yauk CL, Nong A (2013b) Incorporating new technologies into toxicity testing and risk assessment: moving from 21st century vision to a data-driven framework . Toxicol Sci 136:4-18. doi: 10.1093/toxsci/kft178 [doi]

Webster AF, Chepelev N, Gagne R, Kuo B, Recio L, Williams A, Yauk CL (2015a) Impact of Genomics Platform and Statistical Filtering on Transcriptional Benchmark Doses (BMD) and Multiple Approaches for Selection of Chemical Point of Departure (PoD) . PLoS One 10:e0136764. doi: 10.1371/journal.pone.0136764 [doi]

Webster AF, Zumbo P, Fostel J, Gandara J, Hester SD, Recio L, Williams A, Wood CE, Yauk CL, Mason CE (2015b) Mining the archives: a cross-platform analysis of gene expression profiles in archival formalin-fixed paraffin-embedded (FFPE) tissue . Toxicol Sci. doi: kfv195 [pii]

Wignall JA, Shapiro AJ, Wright FA, Woodruff TJ, Chiu WA, Guyton KZ, Rusyn I (2014) Standardizing benchmark dose calculations to improve science-based decisions in human health assessments . Environ Health Perspect 122:499-505. doi: 10.1289/ehp.1307539 [doi]

Wu H, Kerr K, Cui X, Churchill G (2009) MAANOVA: A software package for the analysis of spotted cDNA microarray experiments. In: The Analysis of Gene Expression Data . doi: 10.1.1.3.2986

Yang L, Allen BC, Thomas RS (2007) BMDExpress: a software tool for the benchmark dose analyses of genomic data . BMC Genomics 8:387. doi: 10.1186/1471-2164-8-387
